# Aboriginal artefacts on the continental shelf reveal ancient drowned cultural landscapes in northwest Australia

**DOI:** 10.1371/journal.pone.0233912

**Published:** 2020-07-01

**Authors:** Jonathan Benjamin, Michael O’Leary, Jo McDonald, Chelsea Wiseman, John McCarthy, Emma Beckett, Patrick Morrison, Francis Stankiewicz, Jerem Leach, Jorg Hacker, Paul Baggaley, Katarina Jerbić, Madeline Fowler, John Fairweather, Peter Jeffries, Sean Ulm, Geoff Bailey

**Affiliations:** 1 College of Humanities, Arts and Social Sciences, Flinders University, Adelaide, Australia; 2 ARC Centre of Excellence for Australian Biodiversity and Heritage, James Cook University, Cairns, Australia; 3 School of Earth Sciences, University of Western Australia, Perth, Australia; 4 Centre for Rock Art Research + Management, University of Western Australia, Perth, Australia; 5 ARA—Airborne Research Australia, Salisbury South, Australia; 6 Wessex Archaeology, Portway House, Old Sarum Park, Salisbury, England, United Kingdom; 7 Archaeology, School of Humanities, University of Southampton, University Road, Southampton, United Kingdom; 8 Murujuga Aboriginal Corporation, Karratha, Australia; 9 College of Arts, Society and Education, James Cook University, Cairns, Australia; 10 Department of Archaeology, University of York, The King's Manor, York, England, United Kingdom; Max Planck Institute for the Science of Human History, GERMANY

## Abstract

This article reports Australia’s first confirmed ancient underwater archaeological sites from the continental shelf, located off the Murujuga coastline in north-western Australia. Details on two underwater sites are reported: Cape Bruguieres, comprising > 260 recorded lithic artefacts at depths down to −2.4 m below sea level, and Flying Foam Passage where the find spot is associated with a submerged freshwater spring at −14 m. The sites were discovered through a purposeful research strategy designed to identify underwater targets, using an iterative process incorporating a variety of aerial and underwater remote sensing techniques and diver investigation within a predictive framework to map the submerged landscape within a depth range of 0–20 m. The condition and context of the lithic artefacts are analysed in order to unravel their depositional and taphonomic history and to corroborate their in situ position on a pre-inundation land surface, taking account of known geomorphological and climatic processes including cyclone activity that could have caused displacement and transportation from adjacent coasts. Geomorphological data and radiometric dates establish the chronological limits of the sites and demonstrate that they cannot be later than 7000 cal BP and 8500 cal BP respectively, based on the dates when they were finally submerged by sea-level rise. Comparison of underwater and onshore lithic assemblages shows differences that are consistent with this chronological interpretation. This article sets a foundation for the research strategies and technologies needed to identify archaeological targets at greater depth on the Australian continental shelf and elsewhere, building on the results presented. Emphasis is also placed on the need for legislation to better protect and manage underwater cultural heritage on the 2 million square kilometres of drowned landscapes that were once available for occupation in Australia, and where a major part of its human history must lie waiting to be discovered.

## Introduction

An estimated 20 million km^2^ of territory was exposed on the world’s continental shelves during the Last Glacial Period (c. 110,000–10,000 cal BP), 2 million km^2^ around the Australian continent alone, increasing its landmass by a third [1,2]. During this period some of the major transformations of early human history took place, including renewed human dispersals out of Africa into Europe and Asia, development of seafaring technology, palaeoeconomic diversification and intensification including exploitation of marine resources, and entry for the first time into Australia and the Americas, currently dated at c. 65,000 and c. 20,000 cal BP respectively [3–9]. These previously exposed territories on the continental shelf likely harboured favourable environments for hunter-gatherer settlement and dispersal including abundant water supplies, desirable microclimates, ecological diversity, and the additional potential for exploitation of marine resources and seaborne travel along and around the inlets and archipelagos of its palaeocoastlines [10–12]. These conditions in their turn would have created the potential for relatively high population densities and concentrations of archaeological sites compared to the more arid hinterlands that prevailed in many regions of the world during the Last Glacial Period.

Relatively little detail is known about these now-submerged landscapes, their human occupants, their role in patterns of human dispersal and development, or the human impact of a sustained sea-level rise from a Last Glacial Maximum (LGM) low of −130 m at c. 20,000 cal BP to reach the present level at c. 7000 cal BP [13].

New investigations are now under way in many parts of the world to explore the role of the coastal zone in population dispersal, to reconstruct these submerged landscapes and their palaeocoastlines and palaeoenvironments, and to test their archaeological potential [1, 14–29]. However, systematic recovery and investigation of underwater archaeological sites, which is crucial to the evaluation of new hypotheses, is inhibited by powerful and ongoing constraints. These include the unobtrusive or ephemeral nature of hunter-gatherer material culture, limited knowledge of the taphonomic conditions under which sites preserve and are accessible to discovery, and widespread uncertainties that anything remains to be discovered underwater or that this will be sufficiently intact to make a useful and decisive contribution to new knowledge. There are additional hurdles associated with matters of safety and regulatory compliance as well as consideration of costs of working in a marine environment, which can vary considerably depending on location and conditions.

The potential for submerged sites on Australia’s continental shelves has long been recognized [30–32], but the relatively few attempts made to locate such sites have been unsuccessful [33, 34]. In several areas stone artefact sites and quarries, and stone-walled fish traps, have been documented in intertidal zones [35–38]. However, many of these appear to be extensions of land-based sites and activities related to the present-day shoreline, and it has proved difficult to demonstrate that any were features of a pre-inundation landscape occupied at a time of lower sea level. Underwater artefacts recovered from inland freshwater lakes further underscore the potential for survival of submerged sites on the continental shelf [39].

The new results presented here demonstrate the existence of in situ archaeological material on pre-inundation land surfaces associated with periods of lower sea level in Murujuga Sea Country (Dampier Archipelago, Western Australia), resulting from a program of predictive modelling, systematic survey and geoarchaeological analysis, using a suite of remote sensing techniques including satellite imagery, acoustic survey (sidescan and multibeam), airborne topographic and bathymetric LiDAR, and in situ diver investigation.

The aims in this article are: to articulate the rationale and research strategy that has informed the discovery of the underwater sites in this case study region as an exercise in submerged landscape archaeology; to describe the techniques, equipment and sampling procedures used in underwater mapping, site discovery and analysis of finds; to analyse the results with reference to comparative data from sites on land, with particular attention to the taphonomic and depositional histories of the underwater artefacts and the systematic evaluation of alternative hypotheses about whether or not they can be considered as evidence of human activities on a pre-inundation land surface; and to consider the wider implications for archaeological investigations of the continental shelf in Australia and worldwide.

## Regional setting

**Fig 1:**
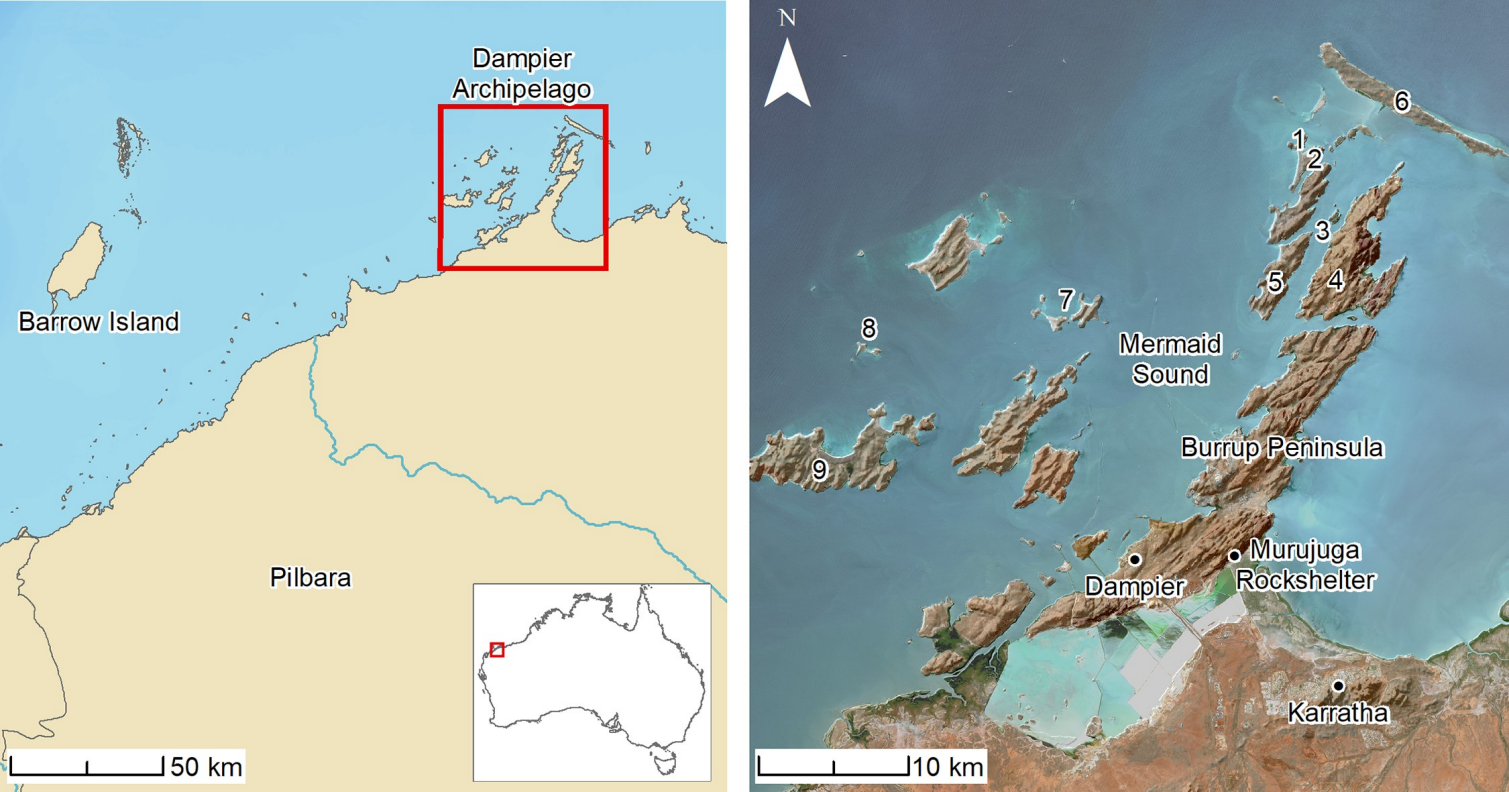
Location maps of the study area and sites referenced in text. 1) Cape Bruguieres Island; (2) North Gidley Island; (3) Flying Foam Passage; (4) Dolphin Island; (5) Angel Island; (6) Legendre Island; (7) Malus Island; (8) Goodwyn Island; (9) Enderby Island.

The Dampier Archipelago (Murujuga) is located in the semi-arid Pilbara region of northwest Australia (Fig 1), and experiences low and variable rainfall averaging less than 350 mm per annum. The archipelago is also situated in one of the most cyclone-prone regions in the world. Thirty-six tropical cyclones crossed the Pilbara coast between 1980 and 2007, and a major cyclone, Cyclone Veronica, passed over the area in March 2019 towards the end of the project’s field campaigns. The potential for cyclone activity to cause disturbance, displacement or destruction of archaeological material in coastal areas is a major factor that needs to be considered when assessing the integrity or otherwise of coastal archaeological sites and their post-depositional history [40]. The timing of Cyclone Veronica provided an unusual opportunity to compare the distribution and condition of archaeological materials before and after the event and to assess the impact of cyclone activity.

The north–south oriented Mermaid Sound separates the archipelago into two island groups. The eastern islands are an extension of the Burrup Peninsula and are formed from 2.7 billion-year-old rhyodacite (also known as granophyre) and gabbros. The western islands are formed from similar-age basalts and andesites [41]. Pleistocene-age aeolianites and cemented beach sediments of mid-to-late Holocene age (calcarenite) are also present around the islands’ coastal fringes and embayments. The former are cemented sand dunes accumulated during earlier periods of high sea level (MIS 5 or earlier) and are characterised by their reddish colour; the Holocene calcarenites are cemented beach deposits including beachrock formed in association with the establishment of modern sea level and are creamy-white in colour.

The igneous geology has eroded into a rough and complex sheet-fractured nubbin terrain with ridgelines of massive boulders (often unvegetated), and valleys which form a rectangular drainage pattern. Freshwater is seasonally available in narrow ephemeral creeks filled by rainfall and in springs [41]. This geology provides abundant and ubiquitous material for manufacture of stone tools and artificial stone structures [42]. It also affords numerous boulders and fractured slabs with surfaces suitable for rock engravings, of which there are estimated to be c. 1 million for the Murujuga rock art province [43]. The slow weathering rates of this geology [44] have created the ideal conditions for the preservation of a human artistic record that could have survived as far back as the 50,000 cal BP that humans are known to have occupied this part of the north-west coast [7].

**Fig 2:**
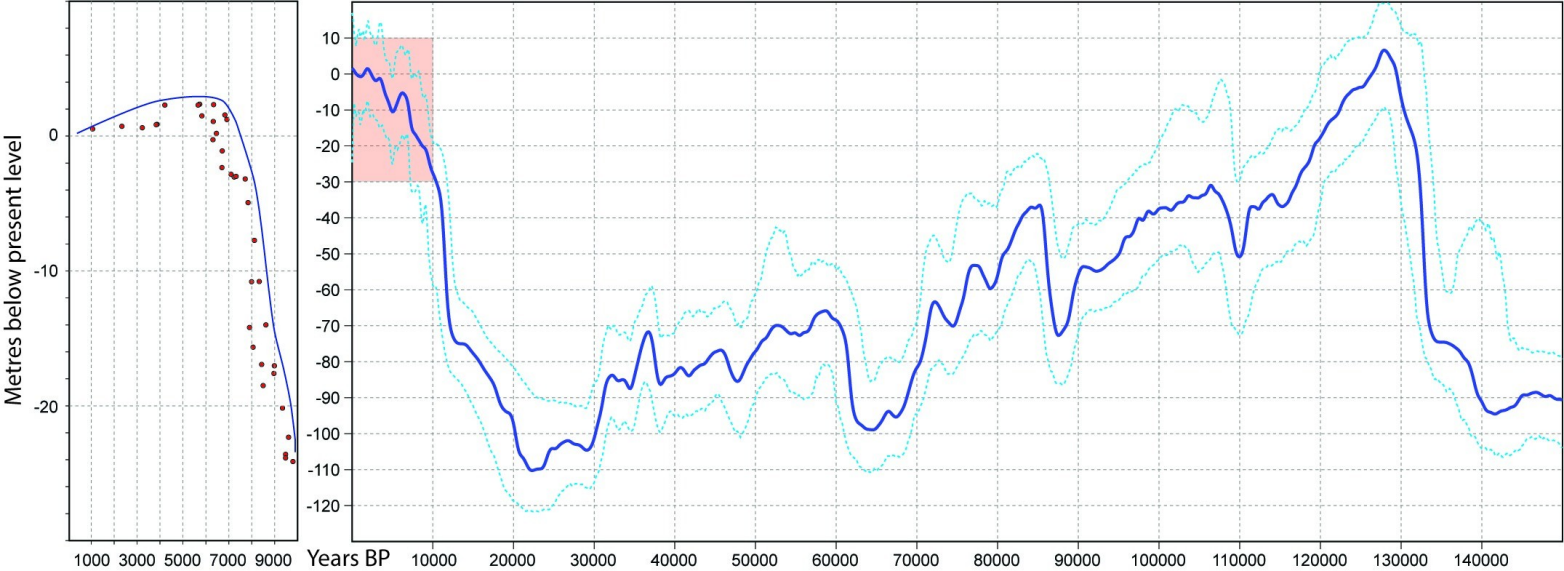
(left) a composite coral growth history from Western Australia, figure modified from [47], blue line (in left insert) represents the minimum age of inundation. (right) Red Sea deep sea oxygen isotope sea level record (blue line) with lower and upper 95% confidence limits (dotted blue line) data from [48].

At the Last Glacial Maximum, the coastline was located 160 km further offshore [45], exposing a gently seaward-sloping coastal plain composed of marine carbonate and siliciclastic sediments, dotted with springs and stream channels, and with fringing mangroves and swamps along palaeocoastlines. Palaeochannels, stranded palaeoshorelines, carbonate reefs, and isolated knolls would have created local relief on the exposed coastal plain. Sea-level rise after the LGM (c. 18,000 cal BP) progressively drowned this landscape, reaching a mid-Holocene highstand of approximately +2 m (MSL) (2 m above modern Mean Sea Level) by 7000 cal BP and subsequently regressing from approximately 5000 cal BP to present sea level (Fig 2; [46]).

The characteristic archaeology of the region is dominated by open-air sites: especially engraved rock art panels as noted above, but also includes: stone tool assemblages; quarries; circular or curvilinear stone structures interpreted as hut foundations or terraces to enhance trapping of sediment; standing stones of probable ceremonial significance; and shell middens, sometimes forming shell mounds up to 5 m thick [42, 49]. Age determinations fall predominantly within the Holocene (the past ten thousand years), but the sequence of rock art styles includes extinct animals, demonstrating a longer history of occupation extending back into the Pleistocene [42, 43, 45, 49, 50]. Rockshelters with stratified and dateable deposits are rare in this type of geology, but one granite overhang on the Burrup Peninsula contains deposits with evidence of occupation extending from 21,000 to 7000 cal BP [51]; while excavations of limestone caves on the more distant Barrow Island have yielded a sequence between 50,000 and 8000 cal BP, confirming the Pleistocene time depth of human activity in the region [7]. The lithic raw materials and food remains found within these sites demonstrate their use as bases for wide-ranging movements into the hinterland and out onto the coastal plain exposed at lower sea level. As sea level rose and the shoreline moved progressively closer, stratified food remains and changing rates of artefact discard show changing patterns of site use and movement across the landscape, increased representation of marine foods, and ultimately abandonment and a reconfiguration of land-use patterns adjusted to the modern coastline [51]. Similarly, rock art motifs show an increase in marine animals with progressive sea-level rise.

### Research strategy and methods

The team deployed a suite of remote-sensing methods to map and interpret the landscape through an iterative process conducted over a series of six field campaigns between 2017 and 2019 [28, 52]. These were designed to identify submarine features of interest and specific targets for closer inspection, to locate submerged archaeological sites for diver inspection, and to recover and analyse geological, geochronological and archaeological samples.

Each of the remote sensing methods and equipment described below has its strengths and limitations, and methods were chosen because of their complementarity and their suitability in combination to provide information at a variety of geographical scales and with varying degrees of resolution and precision. Techniques applied ranged from mapping of topography and bathymetry at a sub-regional scale to high-precision recording of the positions of individual stone artefacts on the seabed. By combining different methods in this way over a series of field campaigns, comparing their results and adjusting subsequent surveys accordingly, the team established a picture of the submerged landscape and identified prospective targets for closer investigation.

This iterative process was designed to take into account five variables: (1) locations likely to have been attractive to the original inhabitants because of proximity to resources such as water supplies and raw materials for stone-artefact manufacture; (2) locations likely to have preserved archaeological materials because of topographic features such as peninsulas and semi-enclosed basins providing protection from destructive wave action and ocean currents, or rock overhangs and cliff lines affording concentration and preservation of accumulated sediments; (3) locations where material was not only likely to have survived sea-level rise but would be sufficiently exposed to be discovered; (4) local knowledge of community members including Traditional Owners and fishermen; (5) accessibility for diver investigation.

Because of limitations on the water depth in which some of these techniques can be applied and their logistical requirements, and taking as a starting point the principle of working from the known (the present-day land surface and its archaeology) to the unknown (the submerged landscape), focus is placed on shallow-water conditions (down to depths of c. 20 m) as a first step into the unknown, and travel distances offshore within relatively easy reach of small support vessels and modern harbour facilities. Investigations at greater depth and further offshore and the search for evidence buried beneath marine sediments pose different challenges and require different technologies and equipment, larger support vessels and different principles of research design and method, a point that is considered further in the final discussion. Fuller details and the results obtained by remotely sensed mapping are presented elsewhere [28] or are in preparation.

All necessary permits were obtained for the described study, which complied with all relevant regulations. The project was conducted under Flinders University ethics approval SBREC7669 and with the approval of the Murujuga Aboriginal Corporation by Circle of Elders vote (19th January 2017). Mapping and sampling was conducted under a Western Australia Department of Parks and Wildlife permit under Regulation 4(1) (5th May 2019). Permission to undertake further analysis of the artefacts was granted by the Murujuga Aboriginal Corporation. Sampled cultural material was repatriated and remains in the possession of the Traditional Owners (see supporting information). The specific techniques and methods which resulted in the successful location of two submerged sites are described in further detail below.

### Airborne LiDAR survey

For onshore and offshore aerial survey and mapping of terrestrial and submarine surfaces at a variety of scales, the team deployed a Diamond Aircraft HK36TTC-ECO Dimona motorglider with two LiDAR systems mounted in under-wing pods: a Riegl Q680i-S (topographic) and a Riegl VQ-820-G (topo-bathymetric), each combined with a tactical grade IMU/GPS system (Novatel SPAN ISA/LCI). A Canon 5D Mk4 was fitted with an EF 24 mm (f/1.4LII USM) lens and co-mounted with the Q680i-S. Point cloud density ranged between 10 and 20 points/m^2^, and data was processed and converted to a Digital Elevation Model (DEM) using the Global Mapper LiDAR module. Airborne mapping offers enormous flexibility in areal coverage and is the only method that can produce a seamless continuum of images and measurements across the interfaces between land, the intertidal zone and the adjacent seabed, including measurements of relatively high precision in both the vertical and the horizontal dimension. Its limitation is that it is confined to shallow water, in the study area region down to water depths of c. 12 m.

### Marine survey

For more detailed examination of sea-bed surfaces and topographic irregularities and exploration of deeper areas of the seabed, a total of 347 linear km covering approximately 150km^2^ were surveyed with an EdgeTech 4125 sidescan sonar system, operated from an 8.5 m support vessel. Survey areas were gridded using Hypack navigation software, with line spacing ranging from 200 m as a minimum to 30 m for higher-resolution coverage of areas of particular interest. Parallel transects were run over selected areas to ensure a systematic and comprehensive coverage of the seabed. Sidescan instrument real-time locations and sonar mosaics were completed in the SonarWiz processing software. In certain areas where more systematic measurements of seabed bathymetry were required, sidescan imagery was supplemented with multibeam bathymetric data acquired from EGS Survey and Australian Marine Services.

### Diver surveys

**Fig 3:**
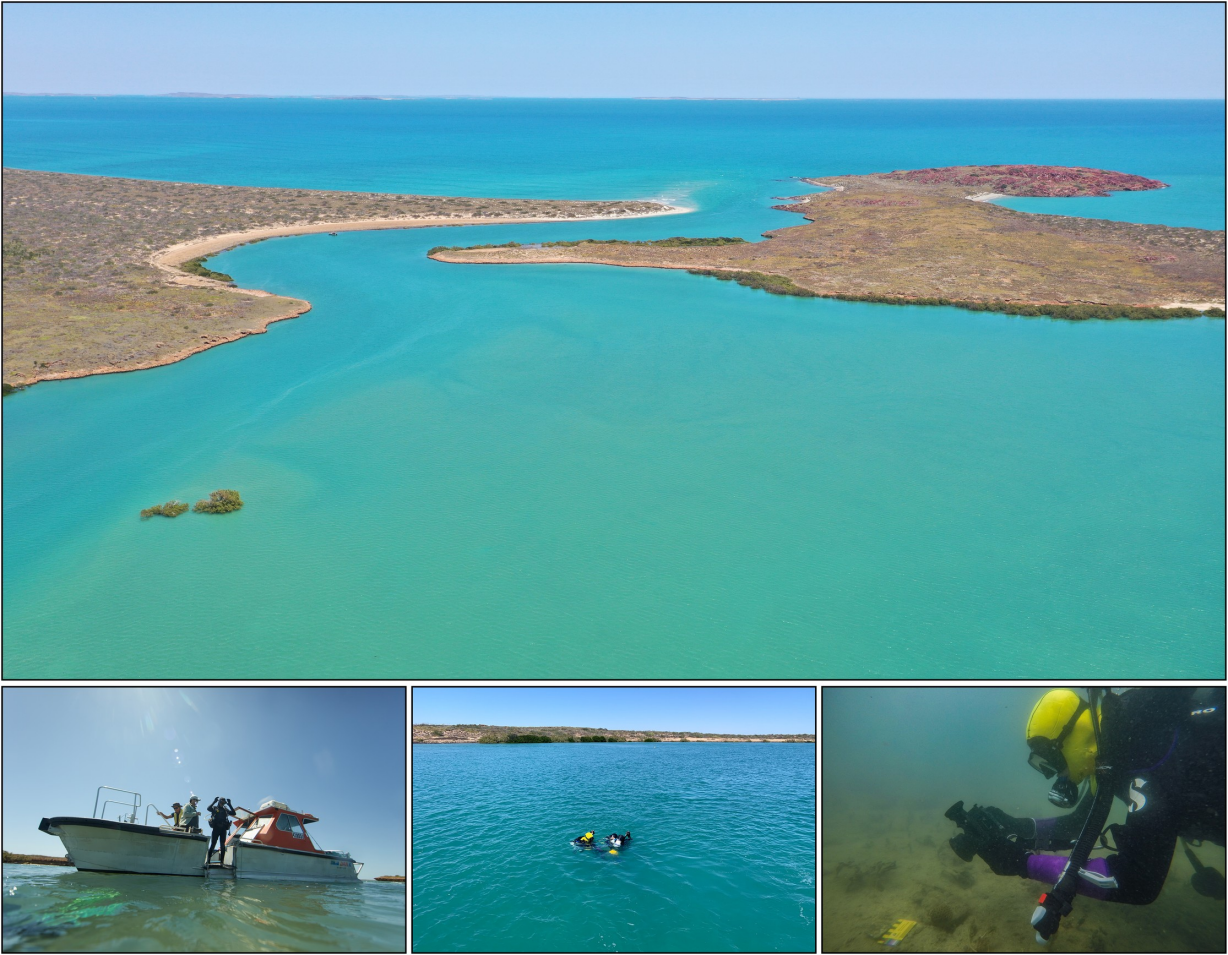
(above) Westward facing aerial view of Cape Bruguieres Channel at high tide (Photo: J. Leach); (below) divers record artefacts in the channel (Photos: S. Wright, J. Benjamin, and M. Fowler).

Once target features of potential significance were identified, the team deployed archaeologically trained divers for closer inspection and sample recovery, using standard safety protocols for scientific diving. Generally, teams of two or four underwater archaeologists worked together on a pre-determined dive plan during any given dive. The navigation system of the dive support vessel was used to position pre-defined survey lines laid out in a GIS with references to aerial and LiDAR basemaps. Survey lines were set using a 100 m leaded line attached to a shot weight with marker buoys on either end. Dive teams carried a marker float with a Garmin eTrex GPS to log location, documented any visible archaeological material and made geological descriptions that included changes in seabed composition (Fig 3). Cameras and GPS were calibrated to enable georeferencing of all photographs. The team explored a number of potential targets in this way before selecting for more detailed investigation the two sites examined here.

### Artefact analysis

Underwater artefacts were examined in situ and recorded with as much information as possible without removal. However, many analyses could not be carried out except in laboratory conditions and a sample of artefacts was removed for that purpose. Each artefact was given a unique accession number, measured, and photographed. The morphological features recorded were raw material type, colour and quality; maximum length, breadth and thickness (in cm); weight (g); artefact type including a range of features specific to flakes and cores; and presence and nature of retouch. Since the amount of marine growth present often meant that characteristic features were obscured, comments on each artefact included an assessment of whether it was a definite, probable or possible artefact (see S1 Table). The nature of the marine growth was also described. This included corals, sponges, bryozoans, tubeworms, foraminifera and coralline algae, the presence and relative composition of which changed relative to depth of submersion. Some of these marine growths were sampled for potential age determination (see below). A selection of these artefacts was hand drawn and captured in 3D using a Sony RX100iii and Agisoft Metashape (v 1.6). The team also applied neutron tomography to selected lithics using the synchrotron at the ANSTO DINGO beam facility in Sydney [53] in order to remove surface marine concretions digitally to reveal the shape of the artefact more clearly.

### Geological sampling

For the analysis of artefact and local igneous rock geochemistry to identify the sources of raw materials used in artefact manufacture, the team used a handheld Portable X-Ray fluorescence (pXRF) device–a Niton XL3t GOLDD+; TestAll Geo, standards in SOIL; 99.995 SiO2 (pure silica standard) using the NIST 2709a soil and sediment standard. This facilitated measurement in the field as well as in the laboratory. In the laboratory, a 5% v/v solution of HCl was used to remove carbonate followed by cleaning with DI water to pXRF selected artefacts from the Cape Bruguieres Channel. For dating and geological analysis of the various aeolianite and calcarenite substrates on which artefacts were located, samples were extracted by hand using a hammer and chisel, or a drill core.

### Radiocarbon (^14^C) dating of marine materials

Accelerator Mass Spectrometry (AMS) radiocarbon age determinations on marine shell and coral embedded in aeolianite and calcarenite were undertaken at the University of Waikato Radiocarbon Dating Laboratory and the Scottish Universities Environmental Research Centre (SUERC) Radiocarbon Dating Laboratory. Surfaces of samples were cleaned, washed in an ultrasonic bath, acid etched in HCl, rinsed and dried. Shells were tested for recrystallization by Feigl staining [54]. CO_2_ was collected and reduced to graphite. Pressed graphite was either analysed at the Keck Radiocarbon Dating Laboratory, University of California [55] or at SUERC [56]. Radiocarbon ages were calibrated using OxCal (version 4.3) [57]. Pre-modern radiocarbon ages were calibrated using the Marine13 dataset [58], with a ΔR of 109±25 [7]. Modern radiocarbon ages (F^14^C%≥100) were approximately calibrated with reference to post-AD 1950 F^14^C regional marine concentrations [59].

### Aerial drone survey

Where aerial photography of higher resolution was required, low altitude drones were deployed for mapping of surfaces and features on land and across the intertidal zone when exposed at low tide. This proved especially valuable in mapping the position and distribution of artefacts and even smaller items in order to assess the degree of disturbance caused by Cyclone Veronica. A DJI Phantom 4 Pro and Mavic 2 were flown with automated flight planning software (Drone Deploy) and employed two survey strategies: single-line transects flown between 75–20 ft above the ground level (AGL); and large-area surveys flown at 82 ft AGL with a frontlap of 75% and a sidelap of 70% to produce a ground sample distance of 1 cm. Images were imported into Agisoft Metashape (v 1.5.4) to create point cloud data using settings for Highest Accuracy, Ultra High Quality and Aggressive Filtering. Resulting dense clouds were filtered to achieve 1 cm point spacing, resulting in data sets containing more than 500 million points. These, in turn, were cropped into five 25 x 25 m sample areas and imported into CloudCompare (v2.11) to facilitate comparison between the two datasets. This approach drastically reduced vertical distortion between the two datasets and allowed for effective quantitative comparison between drone runs conducted over the same surface before and after Cyclone Veronica.

## Results

**Fig 4:**
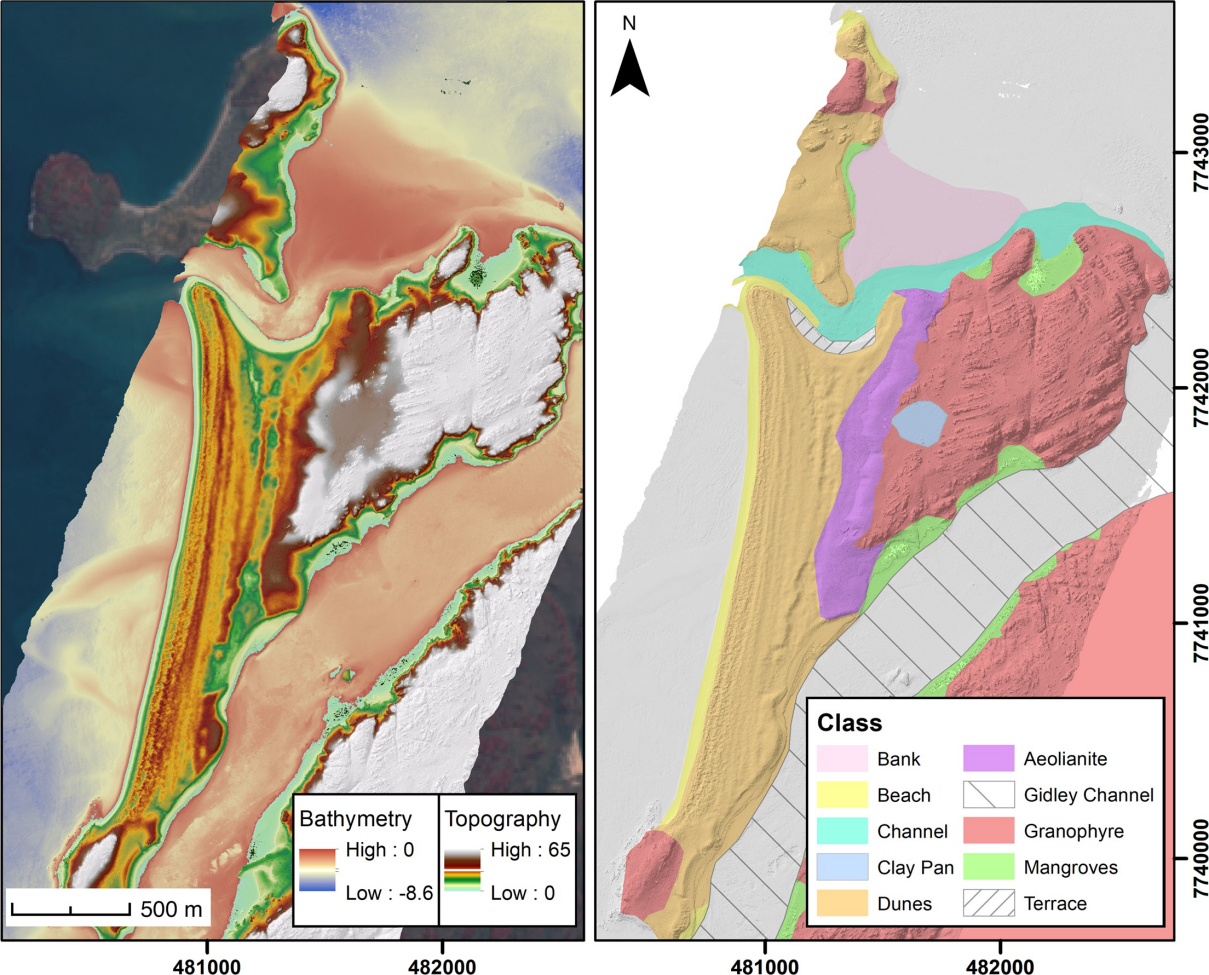
Bathymetry, geology and geomorphology of the Cape Bruguieres Channel and surrounding landscape including part of Cape Bruguieres Island to the north of the channel and North Gidley Island to the south. Note the extensive Holocene dune formations along the west coast of North Gidley Island, the calcarenite terrace on the south bend of the channel, and the mid-channel sill. The highest elevations are on the granophyre bedrock and the Pleistocene aeolianite. Satellite image by Sentinel in the public domain.

Detailed underwater surveys and diver investigations identified two underwater locations in the north of the archipelago assessed to have high archaeological potential, and these were targeted for more detailed investigation. The first is at Cape Bruguieres Channel, where detailed survey by divers identified 269 underwater lithic artefacts to a depth of −2.4 m (MSL) (i.e. 2.4 m below Mean Sea Level) on the relict Pleistocene aeolianite, which forms the channel floor between Cape Bruguieres Island and North Gidley Island (Fig 4). The second find location is in Flying Foam Passage, where a submerged freshwater spring was identified in sonar data at a depth of −14 m (MSL) mid-channel between Dolphin and Angel Islands. Dive survey at this location was limited by logistical considerations but one clearly recognisable lithic artefact was identified and recovered.

Any interpretation of the significance of these two sites must consider them in the archaeological and geomorphological context of the wider landscape. Two other sites in the area are referenced for comparative purposes, one a dry-land site of late Holocene date on a calcarenite beach terrace on the southern shore of the Cape Bruguieres Channel, investigated as part of the current project, the other a cluster of sites in the intertidal zone on the western shore of Dolphin Island facing Flying Foam Passage, which have been previously reported (38).

Because the two new underwater sites are relatively close to these onshore sites (at distances of 10s to 100s of metres), particular attention is paid to the taphonomic and depositional histories of the underwater artefacts and their geomorphic context in order to examine systematically a range of alternative hypotheses that might account for their present position. At one extreme is the hypothesis that all the underwater artefacts are in situ in the locations where they were originally deposited by human activities on a pre-inundation land surface that pre-dates Holocene sea-level rise. At the other extreme is the hypothesis that the underwater artefacts are all in secondary position, having been eroded out from archaeological sites on land on the adjacent shoreline and transported to their present positions by a variety of natural agencies including the action of wind and waves, water currents and downslope movement under the force of gravity.

### Cape Bruguieres channel

This 2.5 km-long tidal channel separates Cape Bruguieres Island (to the north) and North Gidley Island (to the south) and has a maximum width of 150 m and a maximum depth of −2.4 m (MSL) (Fig 4). At its widest point is a U-bend with the outer (southern) shore of the bend bordered by a calcarenite terrace (cemented beachrock). This terrace extends for approximately 600 m around the channel edge, is 80 m wide at its widest point, and is 2 to 2.3 m in elevation, with a gentle seaward slope. The terrace is relatively flat and is positioned slightly above Mean High Water Springs. Elsewhere the channel is flanked by outcrops of igneous rock or Pleistocene aeolianite. Relict Pleistocene aeolianite also forms the channel floor, which is mantled by a thin modern veneer of mobile sands.

The calcarenite terrace is interpreted here as an ebb-tidal sand spit that began to form during and post the mid-Holocene sea-level highstand interval. This interpretation is consistent the terrace elevation and with a radiocarbon date of 2446±65 BP (1791–2141 cal BP; Wk-49709, Table 1) obtained from a shell cemented into the upper surface of the terrace. On the opposite bank of the U-bend, there is only a minor occurrence of this same calcarenite feature.

**Table 1:** AMS radiocarbon determinations for the Bruguieres channel. Five samples on underwater artefacts returned modern ages, which is inconclusive since active marine growth is present on most of the underwater artefacts. The three Pleistocene ages from the channel floor are on marine shells cemented in the rock surface, and are in the range 44.7 to 26.6 ka, which are interpreted as minimum ages. The three modern ages from the channel floor result from dating modern crustose coralline algae, coral or burrowing mollusc shells attached to the underlying bedrock.

Lab. No.	Sample	Method	δ^13^C‰	% Modern Carbon (F^14^C%)	Conventional ^14^C Age (years BP)	Calibrated Age BP (95.4%)	Calibrated Age BP or AD
							Median
Wk-49709	marine shell	14C	1.7±0.4	73.8±0.60	2446±65	1791-2141	1960 cal BP
Wk-50093	coral	AMS	-1.38±0.3	105.9±0.32	0±0	NA	AD 1960s
Wk-50094	marine shell	AMS	-2.34±0.3	104.5±0.33	0±0	NA	AD 1960s
Wk-50095	coral	AMS	NA	101.4±0.31	0±0	NA	AD 1960s
Wk-50096	marine shell	AMS	NA	101.0±0.31	0±0	NA	AD 1960s
Wk-50097	coralline algae	AMS	-0.9±0.3	102.7±0.35	0±0	NA	AD 1960s
SUERC-90023	coralline algae	AMS	-0.5	104.4±0.32	0±0	NA	AD 1960s
SUERC-90024	coral	AMS	-3.3	106.6±0.33	0±0	NA	AD 1960s
SUERC-90025	oyster	AMS	2.3	NA	26,676±75	30,220-30,804	30,508 cal BP
SUERC-90026	marine shell	AMS	3.0	NA	44,745±490	46,326-48,808	47,499 cal BP
SUERC-90030	marine shell	AMS	2.1	107.3±0.25	0±0	NA	AD 1960s
SUERC-90031	marine shell	AMS	2.2	NA	31,605±117	34,665-35,281	34,959 cal BP

According to the tide gauge on Legendre Island, the average tidal range between Mean High Water Springs (MHWS) and Mean Low Water Springs (MLWS) is 3.6 m, and Mean Sea Level (MSL) is the mid-point between them. Hence MLWS is −1.8 m (MSL) and MHWS is +1.8 m (MSL). The maximum tidal range, the range between the highest astronomical tide (HAT) and the lowest astronomical tide (LAT), is +2.3 m (MSL) to −2.4 m (MSL). However, because of local hydrodynamic effects, observed tidal range in the Cape Bruguieres Channel is somewhat smaller; with MLWS at −1.1 m (MSL) and LAT at −1.4 m (MSL). This means that the bed of the channel at −2.4 m is fully submerged at all tidal positions under present-day conditions except on a sill across the middle of the Channel which, situated at a depth of 0 to −1.4 m (MSL), is partially exposed at low tides.

Radiocarbon-dated marine shells (SUERC 90025, 90026, 90031, Table 1) were collected from a marine calcarenite that deposited on top of the aeoloanite on the channel floor. These shells incorporated within the calcarenite are dated in the range 44,700 to 26,600 cal BP. This is interpreted as a minimum date range for radiocarbon-dead marine shell, most likely of Last Interglacial (MIS 5e) age, contaminated with modern carbon introduced by penetration of endolithic marine boring organisms during the past 7000 years. Thus, the channel floor represents an antecedent surface depression in the landscape, which people could have directly occupied at any time after first arrival on the north-west coastal plain. This landscape depression would have become an active tidal channel as soon as the land surface was inundated during the mid-Holocene (i.e. after c. 7000 years ago).

#### Archaeological results

**Fig 5:**
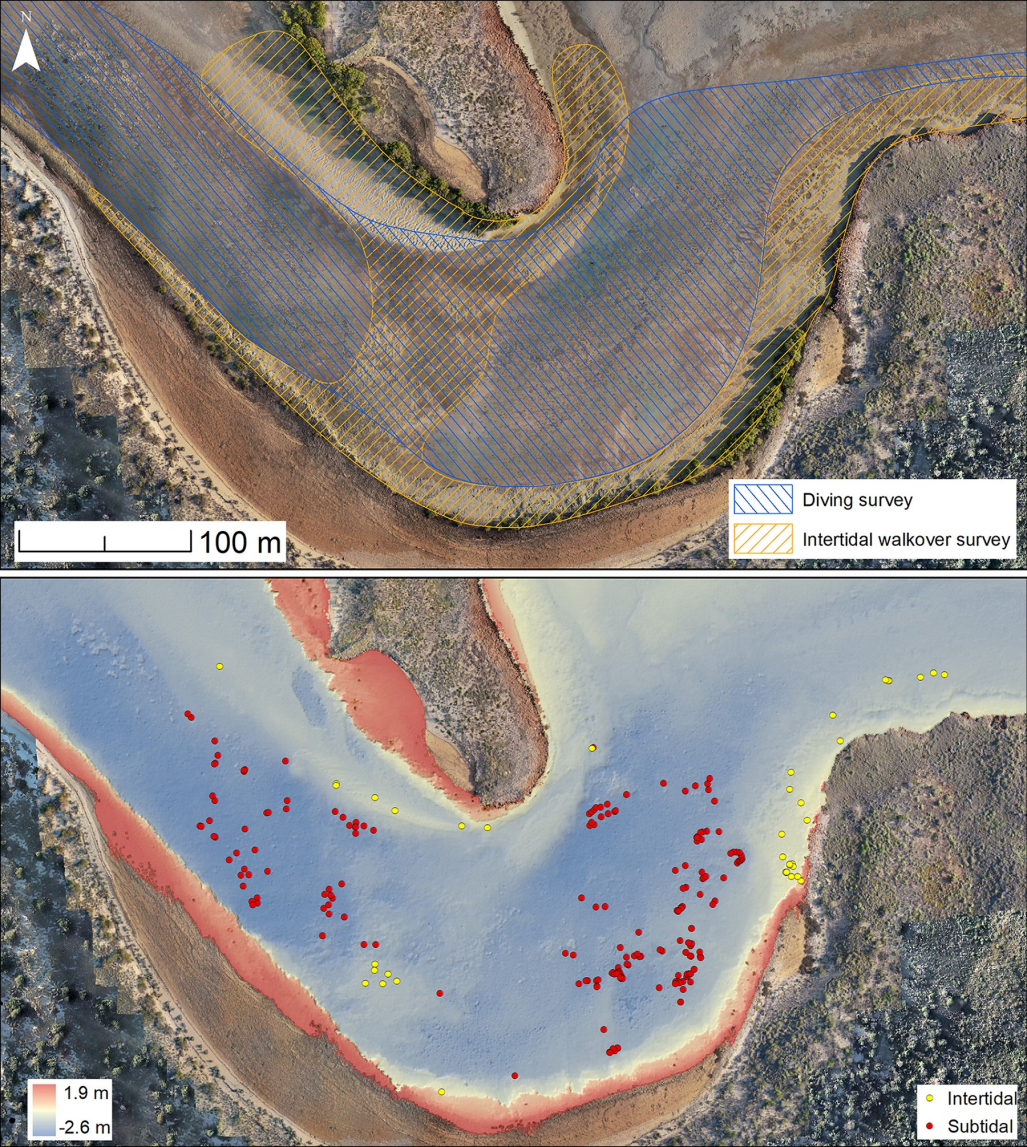
Survey area and locations of underwater and intertidal stone artefacts recorded in Cape Bruguieres Channel. The base photomosaic (above) was taken at Lowest Astronomical Tide (LAT) of 2019. Artefacts are labelled as subtidal (below MLWS) and intertidal (above MLWS and below MHWS). Bathymetric data (below) reflects shoreline at MHWS.

**Fig 6:**
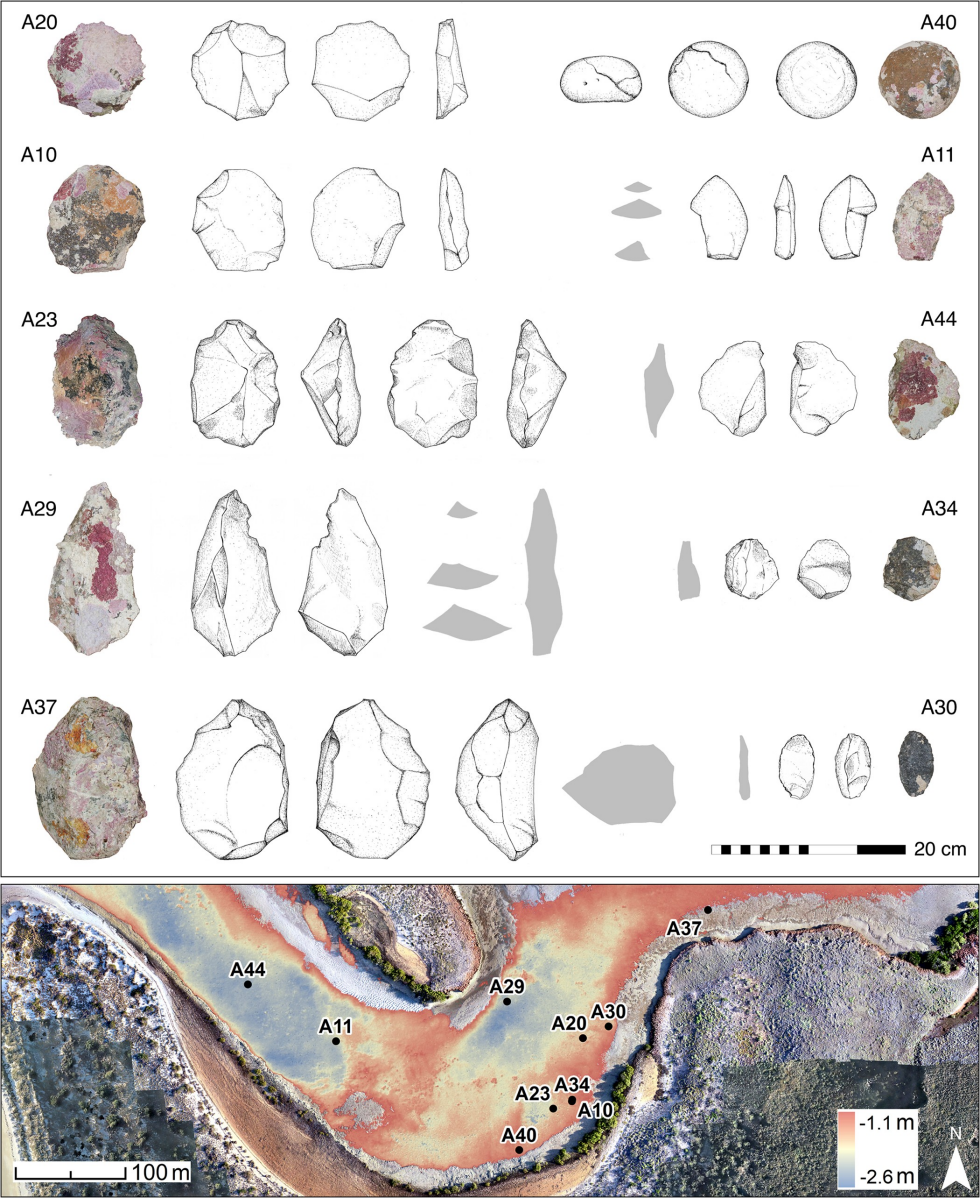
Bathymetric data at Cape Bruguieres Channel (shoreline reflects MSL) with the mid-channel sill clearly visible in the bathymetry. Photos and drawings of a sample of stone tools and their locations on the map by artefact number. Artefacts can be categorised as: Core / Core Tool (A37, A23); Retouched flakes (A11, A29, A44, A30); Flaked tool (A10, A20, A34); Hammerstone/Muller (A40). (Artefact Drawings: K. Jerbić).

Underwater and onshore archaeological surveys have yielded two distinct assemblages of worked stone artefacts. The first comprises 269 underwater artefacts widely distributed across the channel, of which 190 are permanently submerged, with the remaining 79 artefacts in the intertidal zone nearer the edge of the channel or on the mid-channel sill (Figs 5 and 6). The second assemblage comprises 455 artefacts and associated stone structures on the surface of the calcarenite terrace. The artefacts both on land and underwater are made of locally available igneous rocks, and probable quarry sources have been identified to the north on the shoreline of Cape Bruguieres Island. The pXRF analysis shows that almost all the Cape Bruguieres stone artefacts are made of rhyodacite, which forms the main bedrock geology on both North Gidley and Bruguieres Islands. Andesite, commonly found on nearby Lewis and Malus Islands [41, 60] comprises 8% (n = 2) of the terrace assemblage. One of the submerged artefacts was made from a different raw material that has not yet been identified.

The artefacts in the underwater assemblage comprise a range of types including cores and core tools, retouched flakes, mullers and two possible grinding stones. The onshore artefacts on the calcarenite terrace include retouched and unretouched flakes and cores. They tend to cluster around stone arrangements comprising cairns and curvilinear features composed of slabs and fragments of beachrock. They must postdate the formation of the calcarenite and therefore represent Aboriginal activities within the last 2000 years associated with the modern shoreline and its resources, a point confirmed by their association with shell midden deposits of *Tegillarca granosa* (syn. *Anadara granosa*) and Baler shells (*Melo* sp.). There is a more widespread surface scatter of similar materials across the terrace and adjacent dunes, indicating that this whole shore zone was a preferred living area and focus of subsistence activities.

**Fig 7:**
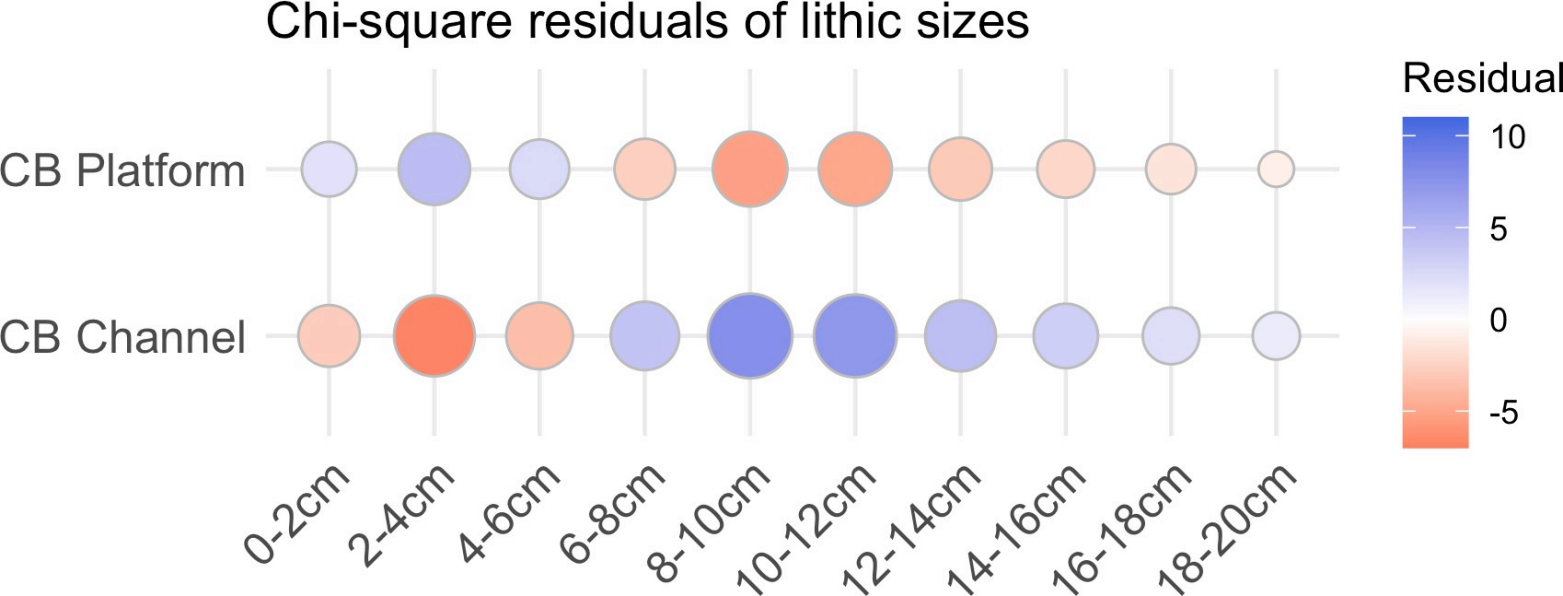
Residual plot for the chi-squared test of artefact sizes from the Cape Bruguieres platform (land) and channel (submerged) assemblages, using the ggcorrplot [64] package in R. Blue circles indicate an over-representation, and red circles indicate an underrepresentation.

**Fig 8:**
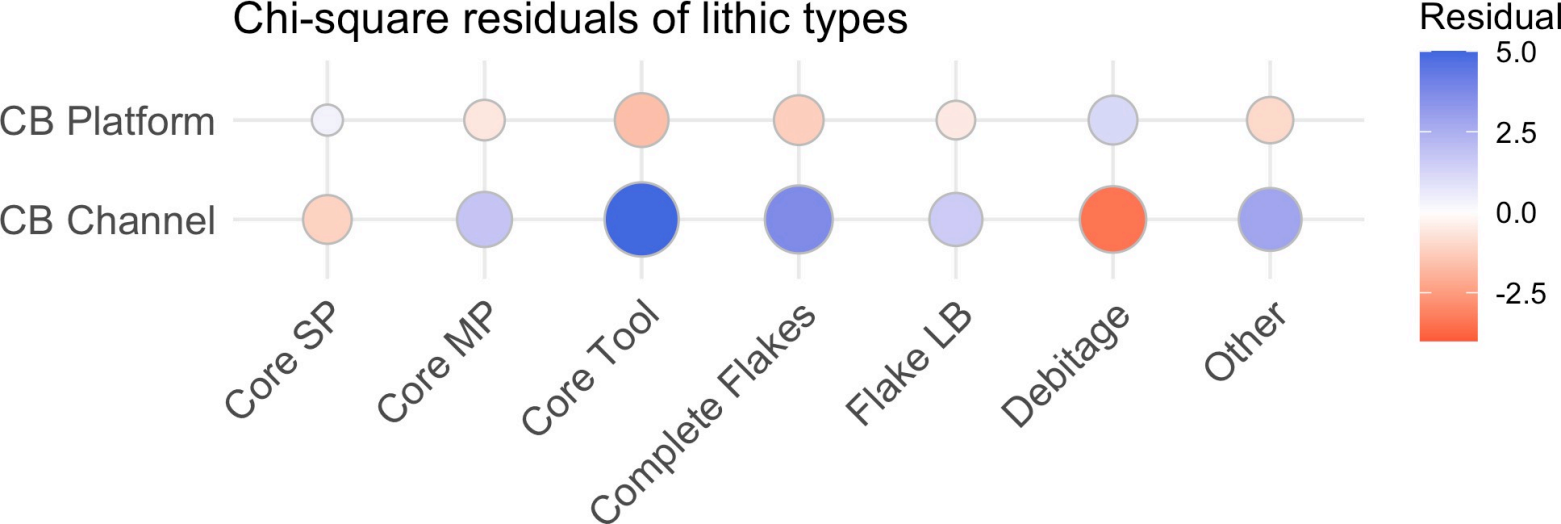
Residual plot for the chi-squared test of artefact types for the Cape Bruguieres artefacts, using the ggcorrplot package [64] in R. Blue circles indicate an over-representation, and red circles indicate an under-representation.

Although there are general similarities in artefact types between the underwater and onshore assemblages, there is also a clear size difference (Table 2). Statistical analysis on the artefacts was performed in the R statistical language using the ‘stats’ and ‘tidyverse’ packages [61, 62]. Size independence testing was focussed on the 0-20cm size range, which represents the vast majority of the Cape Bruguieres assemblage and allows comparison with the Dolphin Island artefact data. A Pearson’s chi-squared test demonstrates the difference in size between Cape Bruguieres assemblages is statistically significant (X^2^ (df = 9, N = 654) = 337.49). The simulated p-value calculated using 2000 replicates of Monte-Carlo random sampling meets the very high threshold of <0.001. The effect size, calculated using Cramér’s V is 0.71, which demonstrates this difference is strong (<0.5) and practically significant [63]. The same test was done for differenced in artefact types, which also demonstrated a statistical significance (X^2^ (df = 6, N = 351) = 72.27, simulated p<0.001) with a moderate to strong effect size (V = 0.45) [63]. The submerged artefacts are more massive with more core tools and generally thicker platforms and less evidence of knapping debitage, in contrast to the terrace assemblage, where artefacts in the 2–6 cm size category (based on measured maximum length) dominate and the larger sizes are far fewer (Figs 7 and 8).

**Table 2:** Summary data of artefact size categories showing the comparison between the submerged Cape Bruguieres channel (underwater, including intertidal) assemblage where artefact size was confidently recorded (n = 210; note: size determinations were not possible for the remainder of the assemblage which was left in situ and where adequately scaled photos were not recorded) and the Cape Bruguieres terrace (onshore) assemblage (n = 445). Of the underwater artefacts, 45 were collected and measured, with the remainder photographed in situ with a scale card.

	0–2 cm	2–4 cm	4–6 cm	6–8 cm	8–10 cm	10–12 cm	12–14 cm	14–16 cm	16–18 cm	18–20 cm	20–22 cm	22–24 cm	40–42 cm	42–44 cm
Bruguieres Channel	0	18	30	44	52	37	13	7	3	1	1	2	1	1
Bruguieres Terrace	25	228	154	34	7	1	0	0	0	0	0	0	0	0

#### Taphonomic and depositional history

Given the close proximity of the underwater and onshore artefact assemblages at Cape Bruguieres Channel, it is critical to address the relationship between the two assemblages. Are they of different ages referring to two separate episodes of activity separated by a gap of at least 5000 years, where the underwater assemblage refers to activity in a shallow dry valley at a period of lower sea level, and the onshore assemblage refers to activity on the modern shoreline? And if that is so, is the proximity of the two assemblages simply a coincidence resulting from the vagaries of underwater site preservation, visibility and discovery, or is it due to the relative attractiveness of this locality but for different reasons at different sea-level positions? Alternatively, are the assemblages of similar age that refer to essentially the same episode of activity, where the underwater material has been eroded out from the calcarenite terrace by wind and wave disturbance and displaced and re-distributed under water by wave action and water currents?

To answer these questions, it is necessary to identify and evaluate three alternative hypotheses regarding the depositional history of the underwater assemblage:

- It is in situ and therefore significantly older than the onshore assemblage and associated with the channel area when it was a terrestrial land surface;
- It is a re-deposited collection of artefacts that have been transported by natural agencies from the archaeological site on the adjacent shoreline and is therefore of similar age and culture to the onshore assemblage;
- It is a lag deposit representing heavier material originally deposited on a Holocene dune feature that filled the channel in the final stages of sea-level rise, followed by erosion of the finer sediment fraction by tidal action, leaving in place the heavier materials, primarily the stone artefacts. On this hypothesis, the artefacts might be of any age within the period of Holocene high sea levels during the past 7000 years.

It is necessary to consider hypotheses (a) and (b) together since evidence against one is by default evidence for the other. Four relevant points are identified below.

1 None of the underwater artefacts show any evidence of rounding or edge-damage consistent with fluvial transport or rolling by wave action.

This criterion alone is not conclusive evidence of in situ location because it is possible that artefacts made on hard material such as igneous rock might be displaced by water action over relatively short distances without evidence of such damage, but some of the artefacts are located more than 50 m from the current shoreline, and some edge-damage might be expected in those cases.

**Fig 9:**
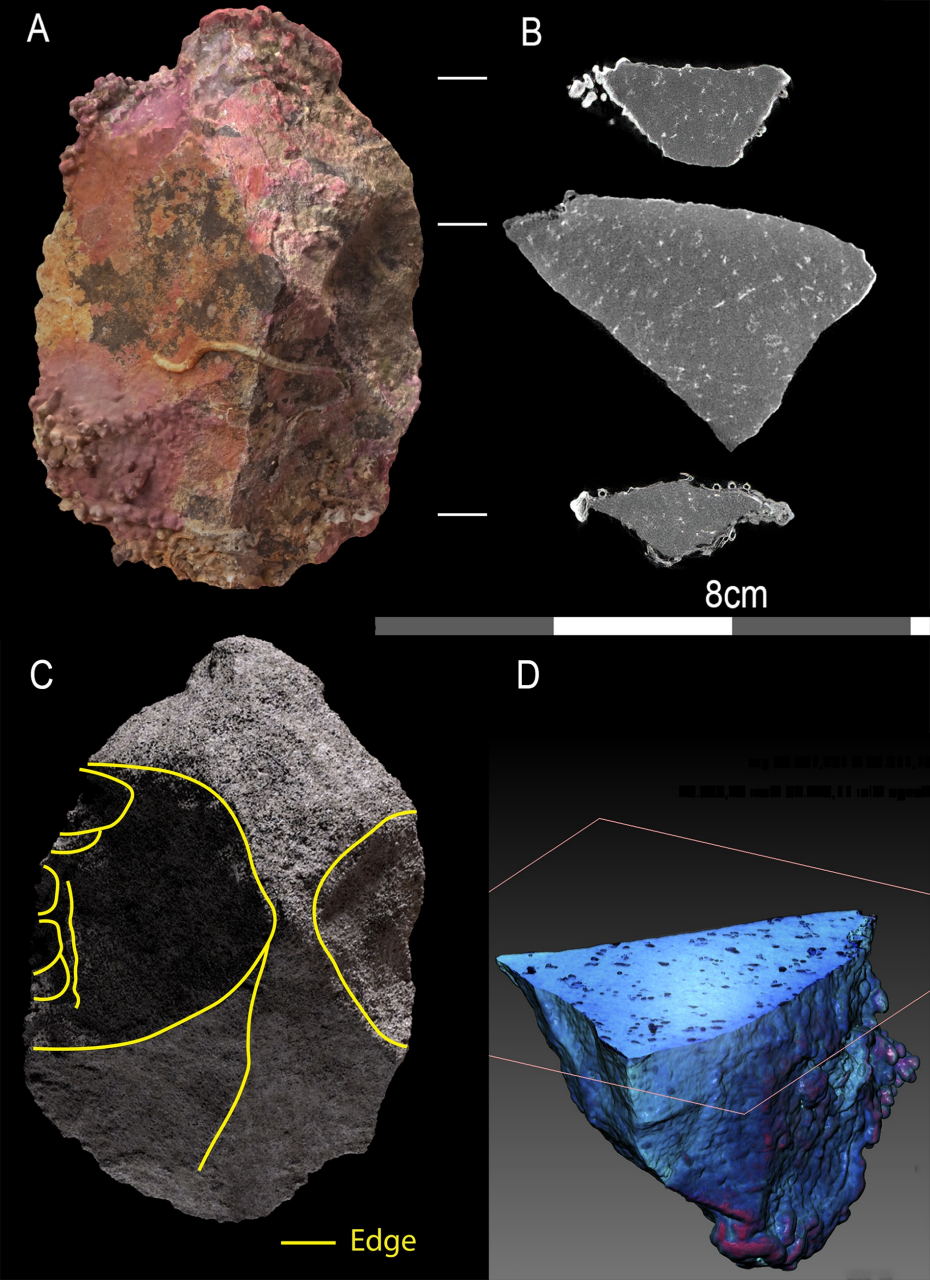
Views of subtidal lithic A23, showing photogrammetric view (A), neutron tomographic slice (B), digitally de-concreted 3D tomography (C) and digitally half-sectioned 3D tomography.

**Fig 10:**
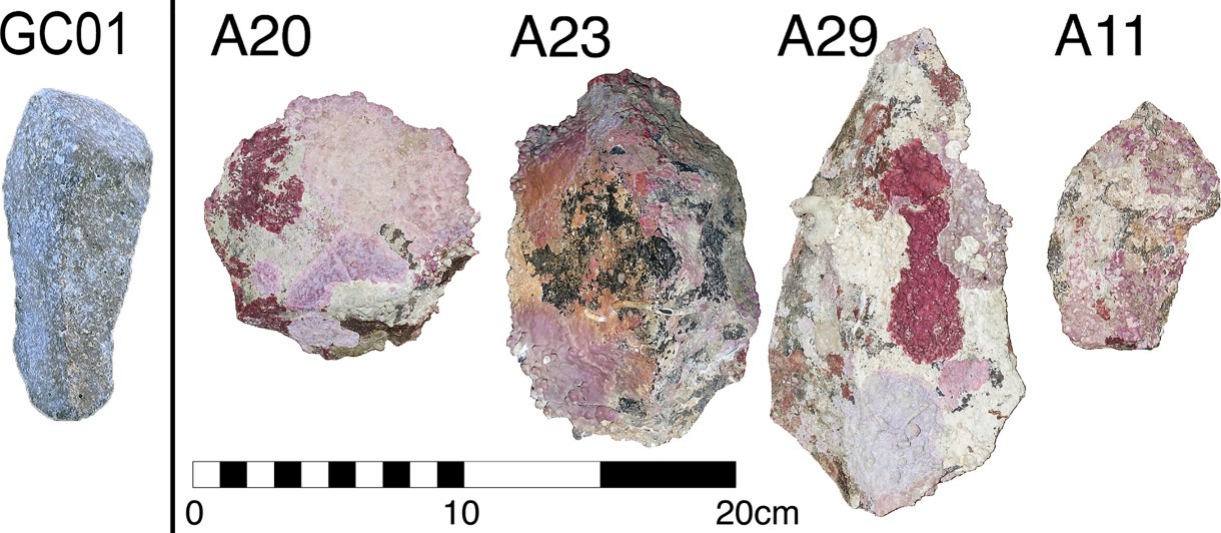
Artefacts recovered under water from Cape Bruguieres (A20, A23, A29 and A11) have well-preserved, acute edges showing no signs of rolling. This is in contrast to material where clear repeated transport and motion has resulted in rolled edges and a lack of marine growth, demonstrated by a possible (but inconclusive) artefact from Goodwyn Island cave (GC01).

Further investigation of this issue was conducted using a synchrotron to examine the edges of 10 of the artefacts collected from the seabed (Fig 9). This demonstrates clearly that beneath the marine growth these artefacts retain acute edges. This is in marked contrast to other material encountered during the surveys by the team, highlighted by a possible artefact of the same material which was found in an intertidal rock shelter on Goodwyn Island, which shows clear evidence of edge rounding caused by water rolling, so much so that confident determination as to whether the object was natural or cultural was not possible (Fig 10).

2 The underwater artefacts are evenly distributed over a large area and at varying depths and show no patterning consistent with the action of tidal currents or waves

**Fig 11:**
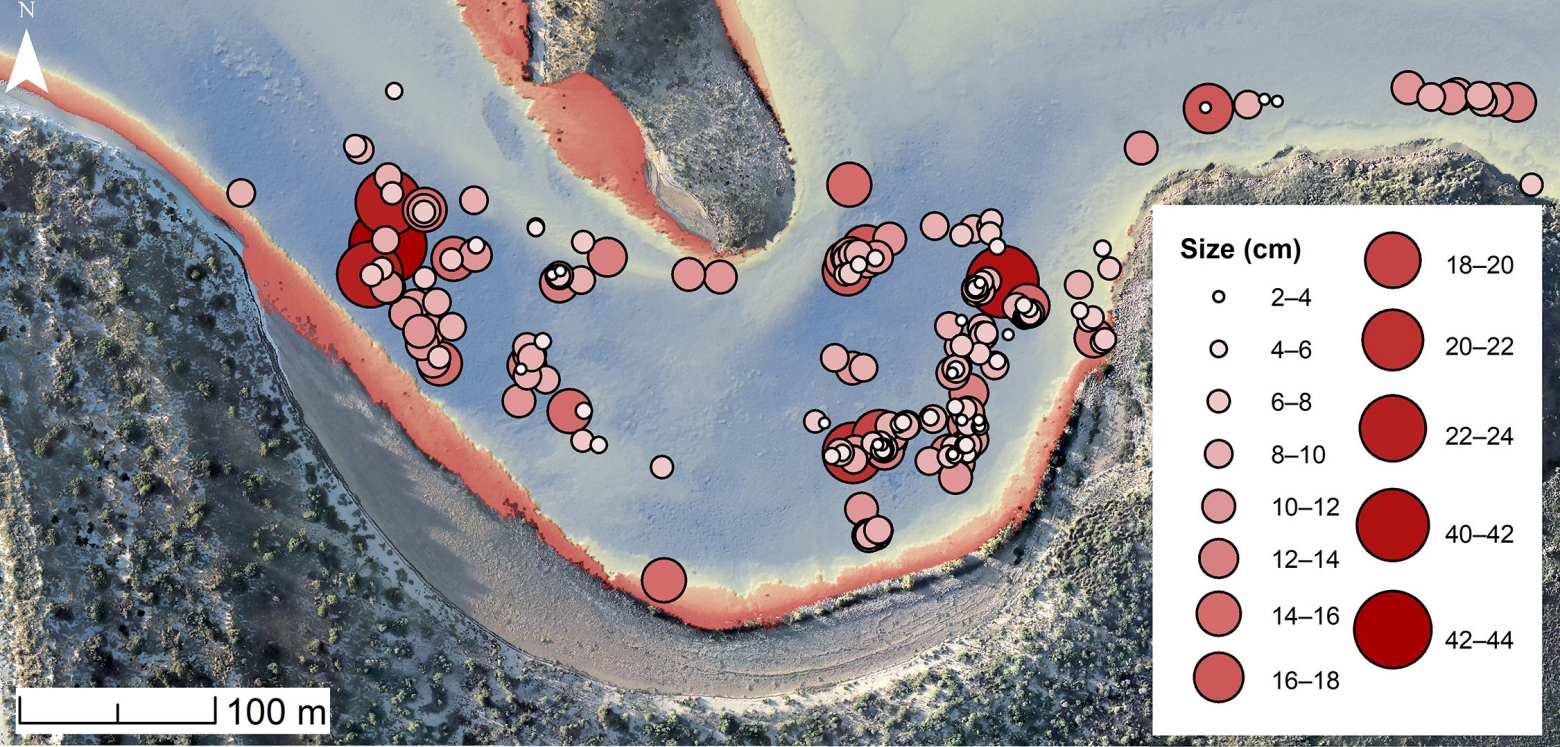
All finds with an associated measurement (as described in Table 2) were plotted on the LiDAR bathymetry in Cape Bruguieres Channel, and the symbols altered to suit a gradient of smallest to largest recorded artefacts. The distribution of the finds indicates no relationship between depth and artefact size.

**Fig 12:**
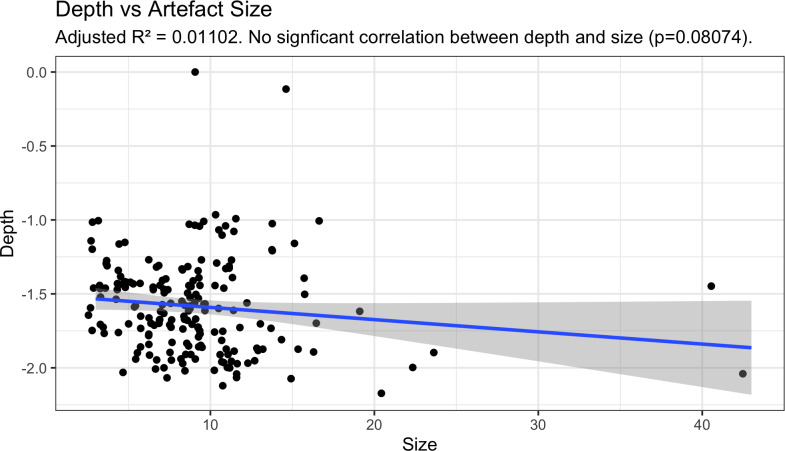
Cape Bruguieres Channel Assemblage size and location data demonstrate the absence of relationship between depth and artefact size. Plotted using the ggplot package in R [62].

There is no evidence of individual size-sorting or linear distributions along particular depth contours consistent with areas of higher-energy currents (Fig 11). A linear model calculated using the ‘stats’ package in R shows a no significant relationship between artefact size and depth (adjusted R^2^ = 0.011, p = 0.08074), with independence comfortably within standard error and the small effect driven by only by a few exceptionally large outliers (Fig 12). Rather their distribution appears random, somewhat like the artefact distribution on the calcarenite terrace (and other terrestrial features), with different-sized artefacts distributed apparently at random in both the horizontal and vertical dimension, and occasionally clustered at specific locations. Had the channel artefacts been derived from the onshore material by high energy wave action, with size differences due to differential sorting by water movement, it would be expected that the largest material would travel over the shortest distances or remain undisturbed while the smaller material would be more likely to travel further offshore. The comparative size analysis (Table 2, Figs 7 and 8) demonstrates that this is clearly not the case and indeed the pattern is the reverse of what would be expected on a hypothesis of differential water transport.

3 There are statistically significant differences in size and morphology between the underwater and onshore artefact assemblages.

The submerged artefacts are larger and more massive than those found onshore, and the flakes have thicker platforms with less core rotation. The absence of the smallest size categories in the channel could relate to differential visibility or sampling bias. However, there is no reason why the rarity of the larger size categories on the terrace could be explained in this way, and this size difference is most plausibly explained by technological differences between the two assemblages. This is further evidence that the underwater assemblage belongs to an earlier time period than the onshore material and is consistent with observed diachronic size variation in dated and stratified onshore sites elsewhere in the wider region [65].

**Fig 13:**
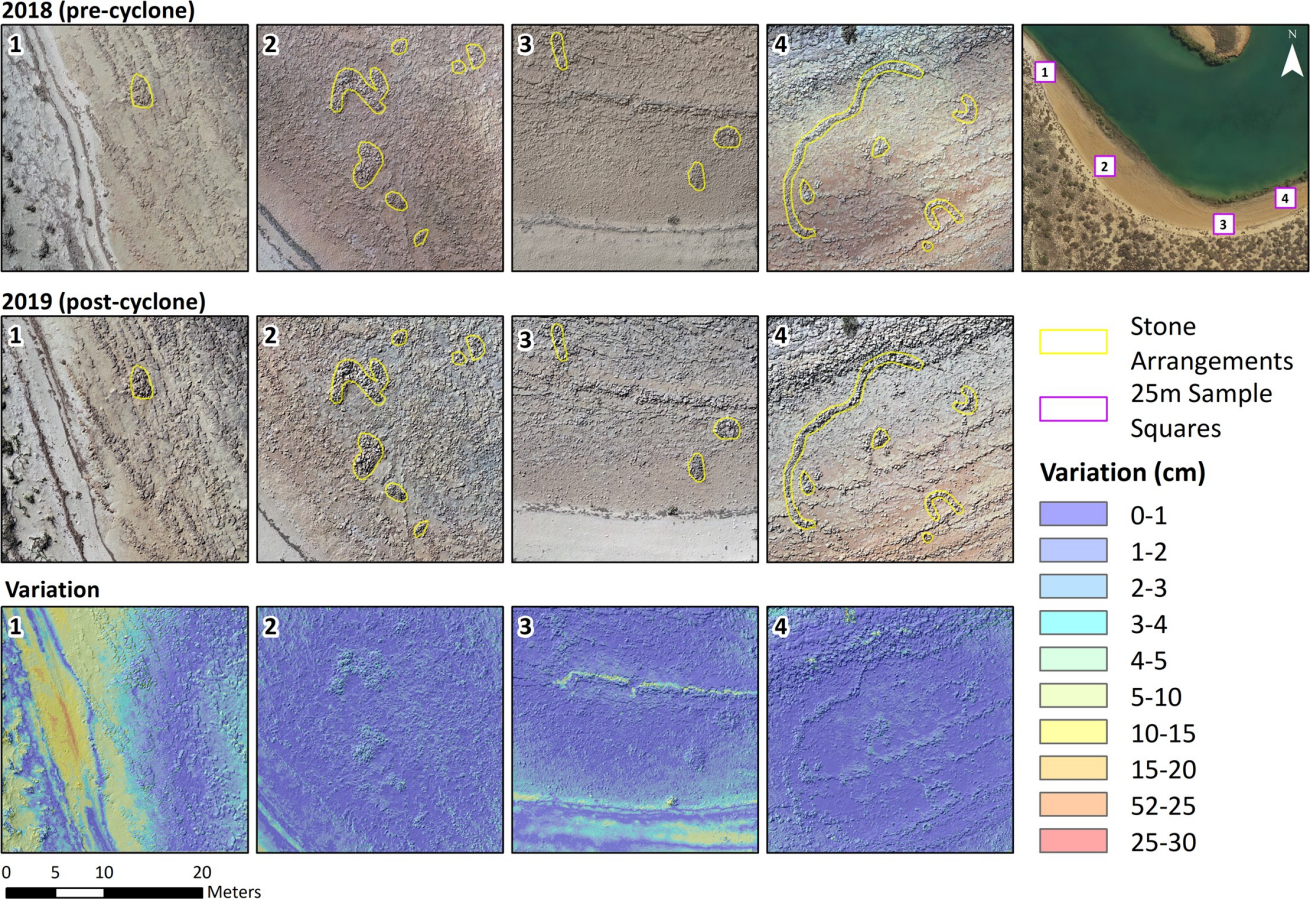
Comparison of the Cape Bruguieres Channel southern beachrock terrace from aerial survey before and after Cyclone Veronica. Area 1 shows the addition of sand in the dune behind the beachrock and a slight change in sediment depth on the platform. Area 3 shows only a slight change in the sediment at the edge of the dune and removal of a line of seaweed in the centre of the image. (Figure: E. Beckett and J. Benjamin). Overview aerial image (top right) reproduced by permission of the Western Australian Land Information Authority.

4 Analysis of the pre- and post-cyclone landscape data shows that the only land surface change was the movement of sand or seaweed across the calcarenite terrace (Fig 13).

Drone imagery was collected over the calcarenite terrace both before and after Cyclone Veronica, a Category 2 cyclone that crossed the Dampier archipelago on 23–24 March 2019. The Legendre Island weather station (located 6 km north of the Bruguieres Channel) recorded a maximum 10-minute mean wind speed of 115 km/h, and a maximum 3-second wind gust of 157 km/h before the weather station went offline [66]. A dGPS-surveyed storm wrack-line at the base of the dune approximately 30 cm above the back of the calcarenite terrace shows that the entire terrace was inundated by a storm surge during the cyclone. Despite this, there was almost no change in the position of the stone features and even small fragments of beach rock remained in situ. This comparison demonstrates that the beachrock terrace and its associated archaeology remained relatively stable throughout this cyclone, and it is thus unlikely that larger artefacts, such as those identified in the channel, would have been transported over the distances required as a result of extreme weather events of this magnitude.

Hypothesis (c)–the lag hypothesis–requires the consideration of the sorts of geomorphological processes associated with Holocene sea-level rise and stabilisation that could have resulted in a two-step process: first the accumulation of sediments to fill all or a large part of the Bruguieres channel and create a living surface for settlement and subsistence activities, and in a second step the erosion of these sediments and the removal of all material except the stone artefacts. The main supporting evidence that could be cited for the first step in this process is the Holocene beach ridge complex along the west coast of North Gidley Island, which formed as sea-level rise stabilised at about its current position, and which now curves around into the Bruguieres Channel culminating in the calcarenite terrace (Fig 4). As part of this first step in the lag hypothesis, it would be necessary to suppose that this beach ridge deposit originally extended continuously along the western shorelines of North Gidley and Cape Bruguieres Islands, blocking the channel that separates them today, and was then breached by tidal action, perhaps during storm surges associated with the mid-Holocene high stand, to create the modern channel, removing the finer sediment fraction and leaving the heavier lithics to deflate onto the underlying Pleistocene substrate.

There are three difficulties with this hypothesis. First, there is no evidence, either from LiDAR DEM or geological surveys, for any remnant of a beach ridge accreted onto the west coast of Cape Bruguieres Island to the north of the channel, which is what would be expected if a beach ridge complex had originally extended continuously across the current channel entrance.

Secondly, there is also no geomorphic evidence on the southern side of the channel for truncation of the beach ridge-calcarenite complex by a channel erosion event. Geomorphic evidence suggests that soon after the area was flooded, the shallow depression between North Gidley and Bruguieres Islands became an active tidal channel with currents mobilising sediments to form an ebb tidal sand spit along the southern edge of the channel. This spit became a permanent feature and allowed the developing beach ridge complex to accrete onto it while the strong flow of tidal currents kept the channel open.

Thirdly, the development of an ebb tidal sand spit on the southern side of the channel and towards its western entrance, and the lack of modern sediment deposition in the channel, suggest that the current speeds are far too strong to allow the accumulation of beach-ridge sediments to fill the channel, as proposed in step one of the lag hypothesis.

Diver observations of maximum current flow during spring tides are a further indication that current speeds are fast enough to transport sand-size particles, inhibiting the accumulation and deposition of substantial sediments of sand and beach sediment. These observations are also evidence that the artefacts are little disturbed by tidal action and could have remained in place over long periods of time in this marine environment.

Further evidence against filling of the channel by soft sediments is the presence of extensive coral communities, which require hard substrates to grow on and cannot tolerate burial by sediment.

The protected environment of the channel, in the sense that it is protected from the full force of wave and surf action acting on a shoreline directly facing the open sea, the lack of cyclone modification, the quantity, condition and random size distribution of the underwater lithics with respect to natural agencies, the significant size and morphological differences between the underwater and terrestrial lithic assemblages, and the geomorphological evidence, all argue in favour of cultural material that has not substantially moved from its original in situ deposition on a Pleistocene land surface. It is not possible to rule out some slight movement of material, or the possibility that some of the artefacts on the inner margin of the intertidal zone have been displaced from the immediate shore edge, but any such movements appear to have been of very limited extent. All the available evidence rules out any hypotheses arguing for wholesale secondary deposition given the state of current knowledge of coastal geomorphological and climatic processes, and rules in favour of the hypothesis that the underwater assemblage is largely in situ on a pre-inundation landscape and belongs to a much earlier time period and an entirely different pattern of landscape use than the archaeological site on the calcarenite terrace.

Turning to consideration of the different patterns of land use associated with the two assemblages, the attractions of the site on the calcarenite terrace are clearly related to the present-day shoreline with its access to marine resources including shellfish, the remains of which are preserved along with the artefacts. The attractions of the Bruguieres Channel site in a pre-inundation landscape are less obvious. If late Pleistocene or early Holocene in date, the site cannot relate to the marine resources of a contemporaneous palaeoshoreline, which would have been some kilometres distant and perhaps much further. The more likely attractions relate to the topographic and geological conditions at what would have been the boundary between the exposed coastal plain extending to the west at lower sea levels, and the outcropping of the Archean bedrock geology to the east. For late Pleistocene or early Holocene Aboriginal communities living on the exposed coastal plain and its palaeocoastlines, the Bruguieres area would have been the nearest source of raw material for making stone artefacts, and therefore an obvious target during seasonal movements into the hinterland.

Moreover, the relatively narrow and shallow swale represented by the pre-inundation Bruguieres channel, located between more elevated rocky terrain immediately to the north and the south, with the more hilly and complex volcanic terrain of the Dampier Ranges to the east, would have afforded ecological and tactical advantages in accessing local patches of sediment, water and plant food, and animals moving through the wider landscape. However, the immediate attractions of the specific locations where individual artefacts were deposited might relate to ephemeral and localised features, such as temporary fireplaces, that have left no surviving trace on the surface of the seabed.

### Flying Foam Passage: A submerged freshwater spring

**Fig 14:**
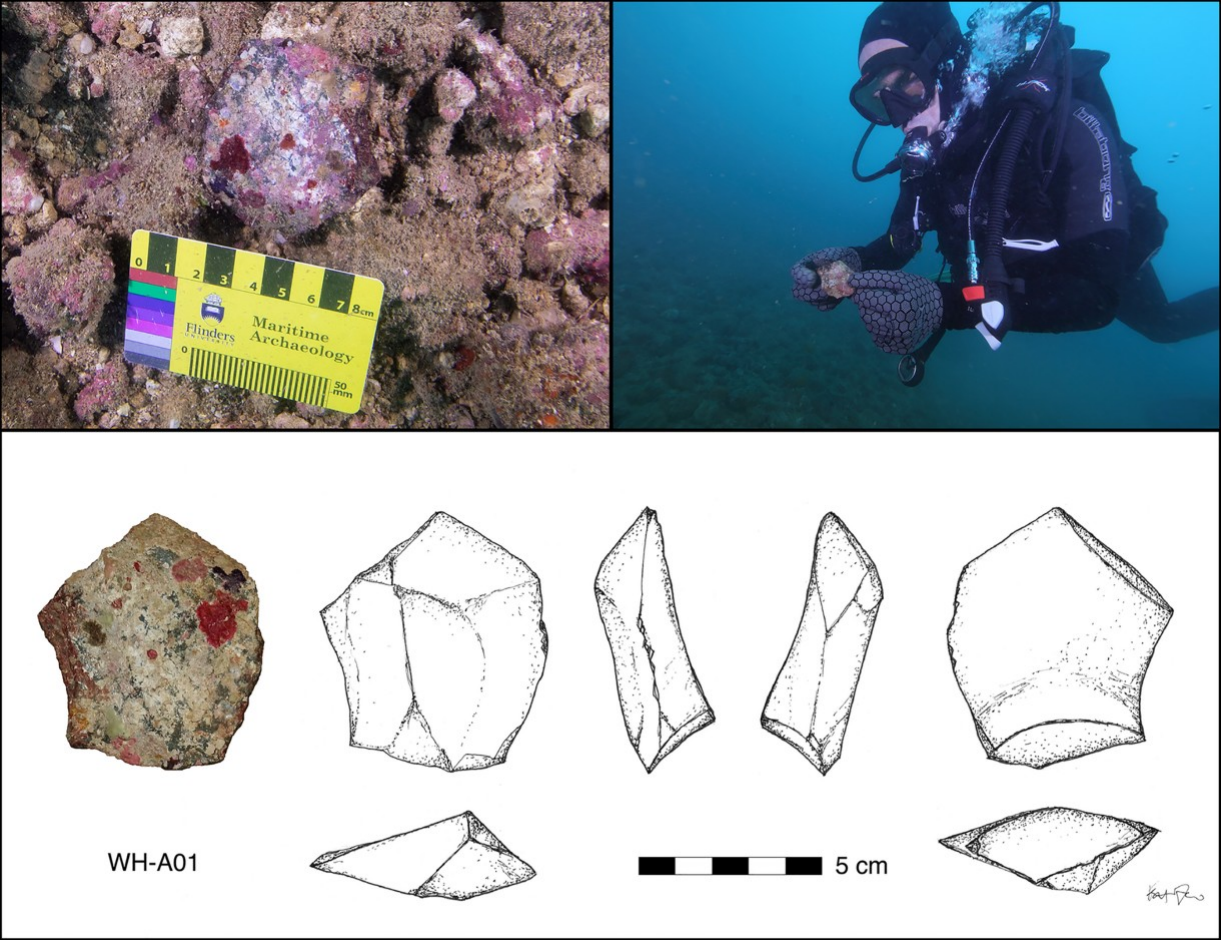
A single artefact was recovered from Flying Foam Passage (WH-A01) where additional material may be present rendering the area a high priority target for future investigations. Inspection below marine encrustations reveals a clear striking platform, a bulb of percussion and dorsal ridges. (Photographs: H. Yoshida; C. Wiseman; Drawing: K. Jerbić).

A second submerged find spot was located within a seabed depression located at a depth of −14 m (MSL) along the central axis of Flying Foam Passage, approximately 600 m west of the outer edge of an intertidal site located on the western shoreline of Dolphin Island [38]. The submarine depression measures 50 m wide x 80 m long (Fig 14). It is one of several locations along Flying Foam passage inspected by divers. The channel floor around the depression is formed from limestone rather than crystalline bedrock, with deep erosional notches cut into the sides of the depression. Multibeam and sidescan sonar imagery over the wider area demonstrate that this is an isolated depression and not part of a fluvial channel flowing through Flying Foam Passage during periods of lower sea level. Therefore, one can confidently conclude that these erosional notches could only have formed through the presence of standing water forming a permanent or ephemeral spring or billabong. Extensive cobble/boulder fields are found around the perimeter and base of this submarine freshwater spring but are almost completely covered in encrusting marine growth, hampering the differentiation between worked lithic artefacts and natural stone. One confirmed lithic artefact was located and recorded from the floor of the depression on the final day of the expedition in this area and is typologically comparable in size, shape and manufacturing technique to the artefacts from Bruguieres Channel. Given the location of this find at a distance of at least half a kilometre from the nearest shoreline and the reconstruction of the palaeotopography, the condition of the artefact, and the direction and relatively weak velocity of current flows once this depression was inundated by sea-level rise [67], it is highly unlikely that this artefact arrived at its present position as a result of being eroded out from a terrestrial site on the nearest shoreline and transported by water action over such a distance. Rather, it is interpreted as evidence of human activity attracted to a freshwater spring in the pre-inundation landscape.

The depth of this find indicates that people could have been present around this freshwater source until the area was inundated by sea-level rise c. 8500 cal BP, giving a minimum age of deposition for the stone tool (Fig 2).

Just how much weight of interpretation can rest on a single artefact is, of course an issue, but the single artefact is the result of limited diving time and the difficulties of identifying artefacts against the background noise of other rocks and obscuring biogenic surface growth, and the details above assure its status as an in situ specimen. In any case, this site, represents an attractive target for more detailed investigation. Perhaps the most significant feature of this find is its association with a submerged freshwater spring. This would have been an obvious magnet for people living on the pre-inundation landscape. These underwater springs (known colloquially as ‘wonky holes’) are known to exist elsewhere on the continental shelf and represent an obvious target for future underwater surveys.

**Fig 15:**
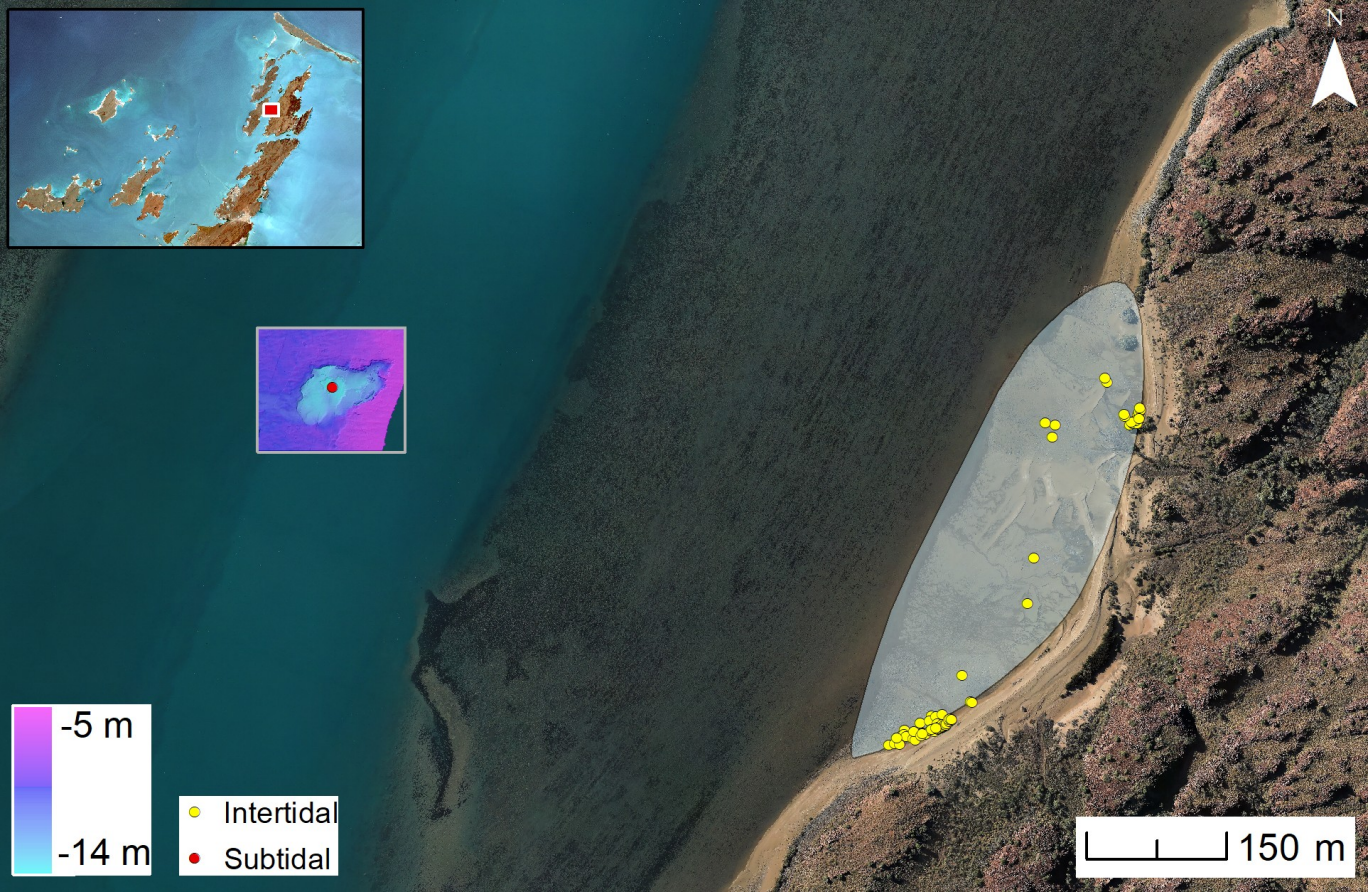
Flying Foam Passage where multibeam sonar imaging of the submerged spring shows the location where a lithic artefact was discovered at −14m (MSL). Distance from the findspot to the Dolphin Island intertidal site is approximately 600 m. Maps used are © Commonwealth of Australia (Geoscience Australia), satellite image by Sentinel (in the public domain).

The adjacent Dolphin Island provides additional context (Fig 15). Here an ongoing project has identified a terrestrial landscape with quarry sites, living areas and rock art panels extending inland from the shoreline, and artefacts on the shore edge and in the intertidal zone (38). These include partially submerged assemblages across the intertidal zone and beach with additional information recovered from the adjacent intertidal mudflats including a rhyodacite flake recovered by coring, found at a depth of 40 cm below the surface of the alluvial sediment.

**Fig 16:**
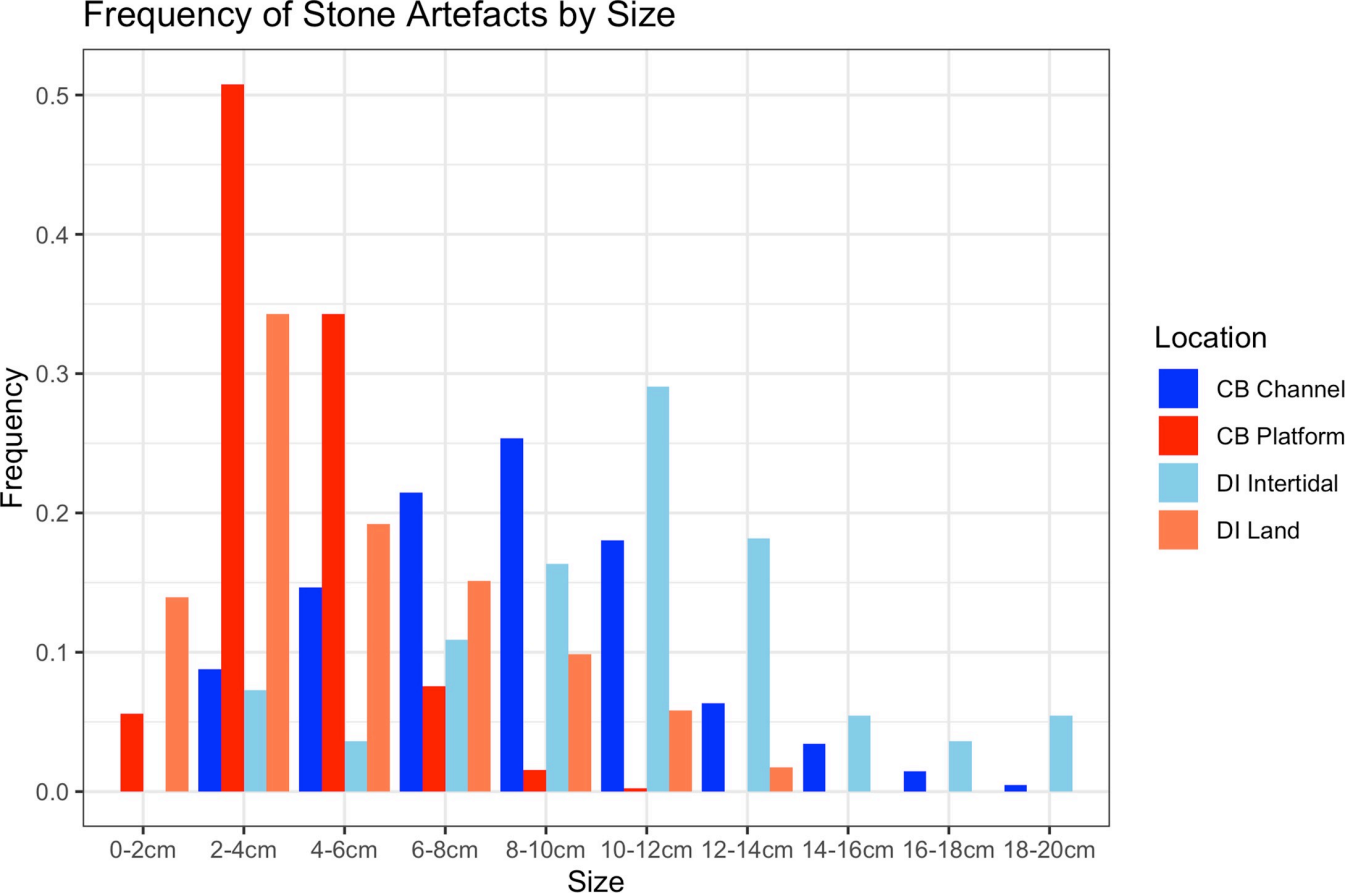
Assemblage histogram (%) of Size categories for the four assemblages from Cape Bruguieres channel (submerged) and platform (land) and Dolphin Island intertidal and land sites.

**Fig 17:**
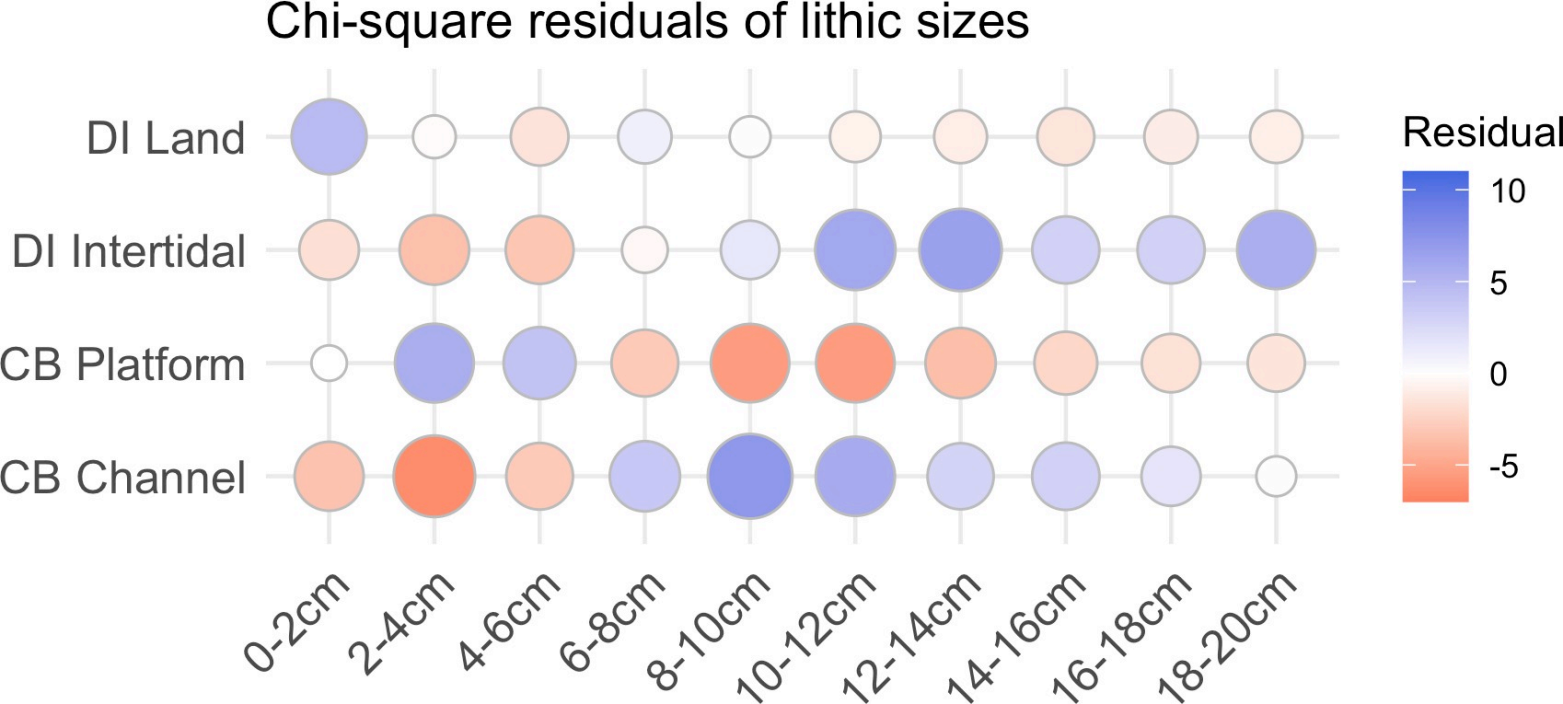
Residual plot for the Pearson’s chi-squared test of artefact sizes from the Cape Bruguieres and Dolphin Intertidal assemblages, using the ggcorrplot package [64] in R. Blue circles indicate an over-representation, and red circles indicate an under-representation. A Pearson’s chi-squared test shows this difference is statistically significant (X^2^ (df = 9, N = 881) = 505.27, simulated p<0.001) with a moderate to strong effect size (V = 0.44) [63].

The potentially early date of this intertidal material is strongly suggested by the size and other features of the artefacts, which show differences between intertidal and inland assemblages on Dolphin Island that are comparable to the differences between the underwater and on-shore assemblages at Cape Bruguiere Channel (Figs 16 and 17). Moreover, the rock art panels show a sequence that includes art styles of an early date belonging to a period when sea-level was lower than present [38, 43].

Investigations to corroborate the early date of the intertidal material and its taphonomic and depositional history are ongoing [68], but the evidence already available clearly reinforces the interpretation of the mid-channel find that this area was an attractive focal point for human activity with a long history of human occupation extending back into a period of lower sea level, and a range of sites with the potential to provide insight into the ways in which local communities responded and adapted to the loss of land and water supplies submerged by sea-level rise.

## Discussion

The past decade has seen interest increasingly focused on the 20 million km^2^ of territory which was available for human occupation on the world’s continental shelves during the low sea-levels of the Last Glacial Period. Exposure of this vast territory and subsequent drowning by postglacial sea-level rise must have dramatically impacted patterns of human population dispersal, cultural interactions and socio-economic development in many parts of the world including Australia. Archaeological evidence from underwater sites is crucial to understanding these processes.

The results presented here demonstrate that underwater cultural material can survive inundation by sea-level rise in an Australian context, and that such evidence can be located and studied using a combination of predictive modelling and an appropriate suite of underwater and remote-sensing techniques. This research also shows how ambiguities over the depositional and taphonomic status of artefacts found in shallow water close to the present-day shoreline can be resolved through archaeological and geoarchaeological analysis and evaluation of alternative hypotheses taking account of known and identifiable climatic and geomorphological processes of coastal disturbance and change. In this respect these results are a first step to investigating the role of submerged landscapes in the peopling of Australia, and an encouragement to conduct similar searches throughout the Asia Pacific region in both shallow and deep water contexts.

Determining the age of cultural material deposited on a sediment-starved seabed, as in Western Australia, remains a major challenge. The Bruguieres Channel site has a minimum age of 7000 cal BP, the artefact adjacent to the submerged freshwater spring in Flying Foam Passage 8500 cal BP, both based on the latest date by when the sites would have been submerged by Holocene sea-level rise SL Figure reference. These submerged sites could be much older since the terrestrial features with which they are associated would have been available since the time of first human entry into Australia. However, no absolute dating methods or other indicators are available that could further constrain the age of these particular sites beyond the limiting dates and minimum ages presented here.

Late Pleistocene and early Holocene coastal archaeological sequences are rare except where offshore islands preserve this time slice and provide a window onto earlier use of the submerged landscape [7,69, 49]. This reinforces the incentive to extend underwater searches for earlier material. The minimum age for the Bruguieres and Flying Foam sites is not especially early, but this reflects the fact that they are sitting on the seabed in relatively shallow water and can only be given a minimum age derived from the regional sea-level curve. Decisive evidence for earlier sites will depend on the search for artefacts in stratified and dateable sediments if they are preserved and can be found, or artefacts on the seabed surface at greater depth and further offshore.

This poses two challenges. The first is the greater logistical complexity and costs of working in deeper water. This requires larger vessels equipped with a full range of acoustic equipment for surface mapping and sub-bottom and seismic equipment for sub-surface mapping as well as facilities for coring and dredging, remotely operated vehicles and, in the event of discoveries that need closer inspection, submersibles and diving teams trained in deep diving techniques and a regulatory compliance environment which allows for scientific diving at those depths.

The second challenge is to develop predictive models that can pinpoint target areas of interest with sufficient precision to focus search and discovery and justify the greater costs of deep-water investigation. One potential target highlighted by these results is submerged freshwater springs. These are known to occur in many locations and at varying depths on the continental shelf, would have been an obvious magnet for human activities, and can be detected by remote sensing techniques. Another potential target is the palaeoshorelines formed at still stands during the Last Glacial, where there is evidence of mangrove swamps and intertidal mudflats, affording access to marine resources and coastal ecotones that would have attracted concentrations of human activity and settlement. These and other possibilities in this region have been identified through a variety of predictive models [28, 67]. Successful deep-water investigations have been conducted in other parts of the world [70, 21, 71] and there is every reason to suppose that they can be applied with equal success in the Australian context.

These findings also reinforce the need for revisions to the existing legislation on the management and protection of submerged Indigenous heritage in the Australian subtidal jurisdiction. Although the national *Historic Shipwrecks Act 1976* has been re-named to be more inclusive as the *Underwater Cultural Heritage Act 2018*, the update only automatically protects shipwrecks and sunken aircraft older than 75 years in Australian waters; however automatic protection does not extend to other categories of other underwater archaeological sites such as submerged Aboriginal sites. This is in contrast to those countries that have signed the 2001 *UNESCO Convention on the Protection of the Underwater Cultural Heritage* (which protects all categories of UCH sites >100 years old), and member states of the European Union, which are required to include submerged landscape archaeology in environmental impact assessments in advance of offshore industrial development [72, 73, 74]. The findings presented in this article highlight that this gap in Australian legislation must be addressed. Experience elsewhere shows that in the wake of such legislation, collaborative arrangements with offshore industries can flourish to mutual benefit, bringing substantial resources and industrial technology to bear on underwater archaeological and palaeoenvironmental investigations [74–77].

Managing, investigating and understanding the archaeology of the Australian continental shelf in partnership with Indigenous Traditional Owners is one of the last frontiers in Australian archaeology, and the results in this article present the first steps in this journey of discovery, in confirming the archaeological potential of Australia’s continental shelves, and in beginning to fill what has remained until now a major gap in the human history of the continent.

## Supporting information

S1 Fig A stone artefact recovered from Cape Bruguieres channel (Photograph: S. Wright). (TIF)

S1 Table Record of all recovered artefacts from Cape Bruguieres channel (CB), Flying Foam Passage (FF), and the Flying Foam Passage submerged freshwater spring (WH1). (XLSX)
