# Correction: Aboriginal artefacts on the continental shelf reveal ancient drowned cultural landscapes in northwest Australia

**DOI:** 10.1371/journal.pone.0287490

**Published:** 2023-06-15

**Authors:** Jonathan Benjamin, Michael O’Leary, Jo McDonald, Chelsea Wiseman, John McCarthy, Emma Beckett, Patrick Morrison, Francis Stankiewicz, Jerem Leach, Jorg Hacker, Paul Baggaley, Katarina Jerbić, Madeline Fowler, John Fairweather, Peter Jeffries, Sean Ulm, Geoff Bailey

In 2019, the Deep History of Sea Country (DHSC) Project team found and published two submerged archaeological sites in Murujuga (Dampier Archipelago) Western Australia [1]. Following publication in 2020, a further discussion has ensued and was published in the journal *Geoarchaeology* by Ward et al [2], with a subsequent response to their critique published by Benjamin et al [3]. This coincided with the project team returning to Murujuga in 2022 to collect further field data and to confirm the nature and context of the two underwater archaeological sites located in the shallow coastal waters of the continental shelf ([4] in press).

This notice seeks to update the *PLOS ONE* readership on the supplemental data underlying [1] now made available and open access; to provide additional methodological information; to address errors in the statistical analyses for Figs 7, 8 and 12; and to provide additional discussion of interpretations.

## Data Availability

With permission from the Traditional Owners, the underlying data for the artefacts reported in this study [1] have now been provided as Supporting Information on this notice (S1 File) and additionally uploaded to a data repository and can be found at: https://doi.org/10.25451/flinders.21907413.v1

Within the underlying data folder, S4_table reports 483 terrestrial artefacts recorded from the Cape Bruguieres (CB) site. This dataset includes the 455 platform artefacts discussed in the original article [1] plus additional data collected at a later date. This supersedes the original data in the article, and it is not possible to identify which of the samples constituted the original dataset of 455 terrestrial artefacts. However, the authors note that the statistical conclusions are identical to those originally published when analyses are repeated on the larger dataset. With this updated data, a Pearson’s chi-squared test demonstrates the difference in size between Cape Bruguieres assemblages is statistically significant (X^2^ (df = 9, N = 687) = 351.31). The simulated p-value calculated using 2000 replicates of Monte-Carlo random sampling meets the very high threshold of <0.001. The effect size, calculated using Cramér’s V, is 0.72, which demonstrates this difference is strong (<0.5) and practically significant. The same test was done for differences in artefact types, which also demonstrated a statistical significance (X^2^ (df = 6, N = 518) = 69.87, simulated p<0.001) with a moderate to strong effect size (V = 0.37).

## Additional Methodological Information

### Tidal data

As stated in the subsection titled Aerial drone survey in the methodology section of [1], “A DJI Phantom 4 Pro and Mavic 2 were flown with automated flight planning software (Drone Deploy) and employed two survey strategies: single-line transects flown between 75–20 ft above the ground level (AGL); and large-area surveys flown at 82 ft AGL with a frontlap of 75% and a sidelap of 70% to produce a ground sample distance of 1 cm. Images were imported into Agisoft Metashape (v 1.5.4) to create point cloud data using settings for Highest Accuracy, Ultra High Quality and Aggressive Filtering.” To add to this, Agisoft Metashape was used to export 2D orthophotos. The orthophotos were georeferenced by the internal GPS of the drone. Default orbit and corrections were used. The datum and height reference is Australian Height Datum (AHD) 2009 on the Australian Map Grid system (in this case using Zone 55) and Australian Geodetic 1984 datum. The elevations of surrounding landscape and neighbouring landmarks have not been determined. These measurements are often incorrect unless taken with highly accurate instruments and processing. It is standard to use high-quality airborne LiDAR to confirm ground-based measurements, especially in inaccessible or remote areas, and this was done for the project through manned aircraft survey. The details of the LiDAR data acquisition are found in the subsection Airborne LiDAR survey: “the team deployed a Diamond Aircraft HK36TTC-ECO Dimona motorglider with two LiDAR systems mounted in under-wing pods: a Riegl Q680i-S (topographic) and a Riegl VQ-820-G (topo-bathymetric), each combined with a tactical grade IMU/GPS system (Novatel SPAN ISA/LCI). A Canon 5D Mk4 was fitted with an EF 24 mm (f/1.4LII USM) lens and co-mounted with the Q680i-S. Point cloud density ranged between 10 and 20 points/m^2^, and data was processed and converted to a Digital Elevation Model (DEM) using the Global Mapper LiDAR module.” This is standard for airborne LiDAR data acquisition, using state-of-the-art full waveform LiDAR and tactical-grade IMU/GPS-systems. For such data, ground-control points (GCPs) are not typically necessary to achieve accuracies of < 10cm margin of error. However, the team did acquire 99 GCPs using Trimble Net R9 dGPS with RTX Satellite subscription, which confirmed that the elevations were correct to within approximately 5cm, and thus no further adjustments were required. The LiDAR and dGPS GCP data can be found freely available at https://doi.org/10.25451/flinders.22641040.v1 Further tidal data collected in 2022 also corroborated the original findings from [1] (the results are in press, [4]).

The Results subsection titled Taphonomic and depositional history refers to diver observations of maximum current flow during spring tides. Fast flowing drift dives through the CB channel were undertaken by divers on 14/09/2019 between 1225–1325 and again on 15/09/2019 between 0855–0925.

### Terrestrial survey

In 2019 a stone structure survey was undertaken by pedestrian transect across the CB platform by three team members. The coverage of this survey was generally 5-10m, denser than the 20m spacing considered “comprehensive” for linear survey in this region [5]. Spatial information was recorded using handheld Garmin GPS units or a Trimble Nomad. Artefacts were observed in varying quantities across the entire platform and a few isolated artefacts were recorded. All artefacts within a 1m radius of each of the 50 recorded structures were recorded. Attribute data (size class, type and raw material) were collected using the CRAR+M FileMaker Pro Recording Form on iPad minis.

Targeted sampling strategies, particularly those constrained by “topographical or man-made features”[6], are common in archaeology. Although the artefact ‘sample areas’ are biased spatially around the structures there is no evidence that this bias would influence the size class data collected (the only attribute being compared between terrestrial and underwater assemblages). The structures are well distributed across the platform and artefact densities were varied within each sample area, with higher densities of artefacts in areas with higher densities of structures. The low structures are made from the same material as the CB platform and there is no evidence that the geomorphic environment immediately adjacent is any different than the rest of the platform. As with the sampling strategy, the size of the sample was constrained, however it was sufficient (more than double the number of channel artefacts) for preliminary comparison. There is no hard and fast numerical or proportional test for the appropriateness of a sample beyond “more is better”, however it is generally accepted that a sample is more robust if you can draw random samples and not dramatically change the statistical result [7]. As noted above, additional artefacts have been added to this dataset (S4_Table in S1 File) since publication of [1] and this increases the dataset by 7%. This did not weaken the statistical result, which would be expected if sizes of the artefacts were biased due to their association with the stone structures.

The terrestrial assemblage at Cape Bruguieres is quantitatively distinctly different to the underwater assemblage: the lithics are much smaller, and the assemblage contains very few artefacts > 8cm, which was the most common size class under water. While the different survey methods might limit the ability for fine-grained comparisons, it could not reasonably explain such a large difference. In the original study, this obvious difference was quantified statistically as a Cramér’s V of over 0.7, defined in the literature as a ‘strong effect (0.5–1 according to Wolverton et al. [8]). To reduce this to a weak effect (Cramér’s V under 0.1, [8]), we would have to suppose that we systematically failed to observe literally hundreds of large artefacts away from structures in our wider survey across the platform (see supplementary R-code for details on this counterfactual). There is scope for a more detailed survey of the platform, to answer finer questions of technology (e.g.[9]), but there is no doubt that there are well-demonstrated and -quantified size differences between the assemblages. The terrestrial assemblage is thus reasonably described as representative and appropriate for comparison with the underwater stone artefacts.

## Errors in data and analyses

One artefact (A15) was incorrectly omitted from the originally published S1 Table in [1], which reported recovered underwater artefacts. The corrected complete dataset for recovered underwater artefacts is provided in Table S3 of S1 File.

There is an error in the analysis underlying Fig 7 of the original article; sample A15 was incorrectly recorded as 10–12 cm instead of 8–10 cm. A reanalysis using the corrected channel artefact data and the updated terrestrial artefact data does not affect the interpretation of the results (please refer to the Data Availability section above for further information). The residual plot for the chi-squared test of artefact sizes from the Cape Bruguieres platform (land) and channel (submerged) assemblages is provided as S2_fig in S1 File.

There is an error in the analysis underlying Fig 8 of the original article; some of the platform type values were omitted. The outcome of a reanalysis using the updated terrestrial artefact data remains that there is significant variation between groups, though there is a slight increase in overrepresentation of core tools, and a slight decrease in overrepresentation of Complete Flakes (please refer to the Data Availability section above for further information).

Fig 12 from the original article accidentally excluded 22 data points. A corrected Fig 12 is provided below. The data that underpin the corrected Fig 12 are derived from S2_table in S1 File. The updated data and figure support the original interpretations and findings.

**Fig 12:**
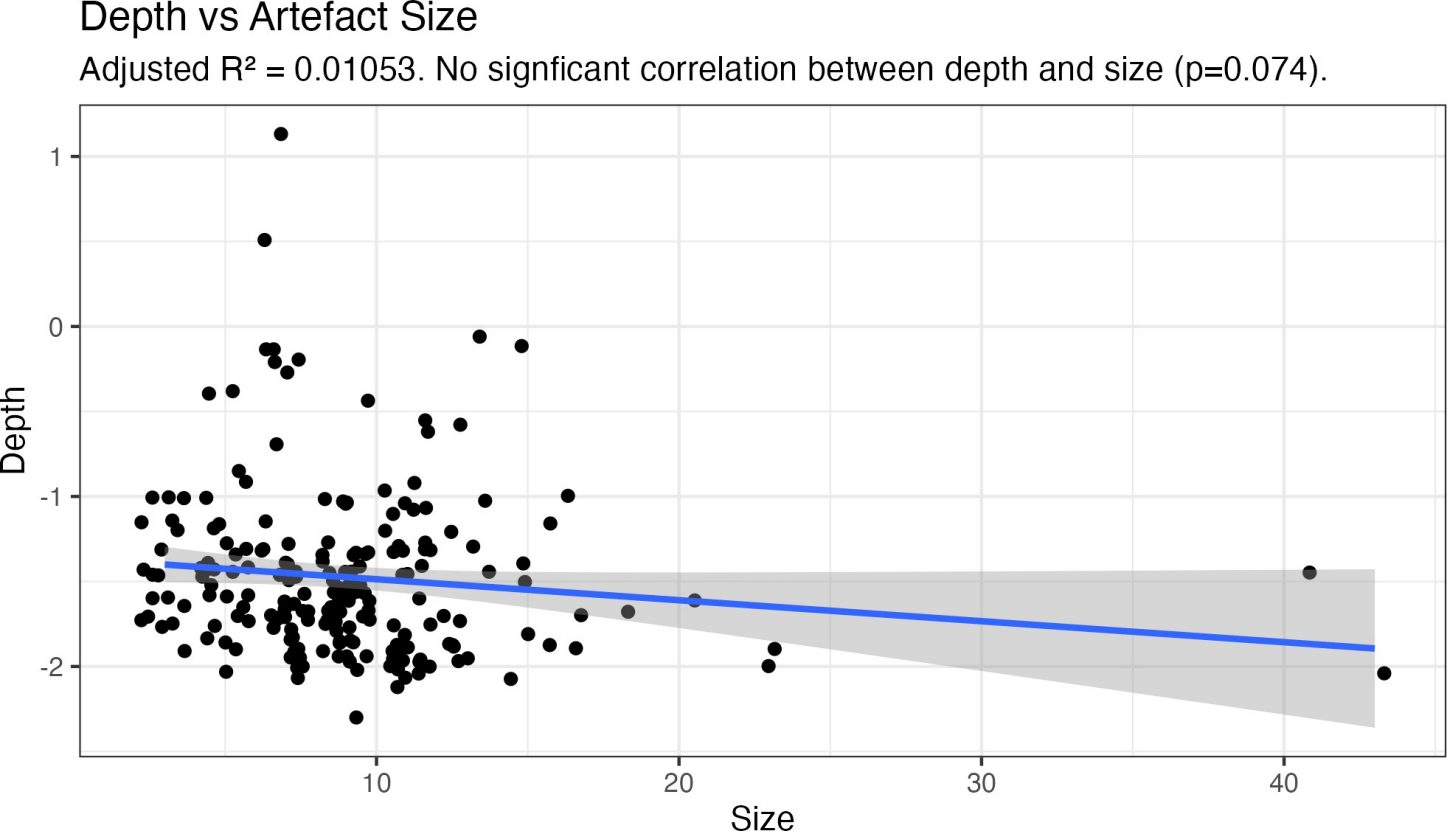
Cape Bruguieres Channel Assemblage size and location data demonstrate the absence of relationship between depth and artefact size Plotted using the ggplot package in R [62].

A member of the Editorial Board has reviewed the updated analyses and advised that the revised data still support the published results.

## Interpretation

In ‘A wet strawman: A response to Ward et al.’ published in *Geoarchaeology* [3] the authors have reiterated the interpretations of the minimum ages of the two sites published in *PLOS ONE* and reviewed why these interpretations remain in place. From Benjamin et al. ([3], pg 3) the authors conclude, “It may well be that we will never achieve 100% certainty about the status of artifacts that are surface finds without means of direct and independent radiometric dating… On the presently available evidence, we reject Ward et al.’s critique and stand by our original interpretations until such time as new and relevant evidence is forthcoming to further confirm or refute them”. This interpretation is further reinforced by additional field investigations in 2022 and the authors have interpreted the available evidence to rule out any hypotheses arguing for wholesale secondary deposition. The authors maintain that the results presented in Benjamin et al [1] demonstrate that underwater cultural material can survive inundation by sea-level rise in an Australian context, and that such evidence can be located and studied using a combination of predictive modelling and an appropriate suite of underwater and remote-sensing techniques. The authors’ current interpretation of the CB Channel site remains consistent with the minimum age of inundation or 7000 BP.

For clarity that the reported conclusions reflect the authors’ interpretations based on the available evidence, the following revisions are made to the text:

In the first sentence of the fifth paragraph of the Introduction, the correct text is: The new results presented here demonstrate the existence of archaeological material deposited on pre-inundation land surfaces associated with periods of lower sea level in Murujuga Sea Country (Dampier Archipelago, Western Australia), resulting from a program of predictive modelling, systematic survey and geoarchaeological analysis, using a suite of remote sensing techniques including satellite imagery, acoustic survey (sidescan and multibeam), airborne topographic and bathymetric LiDAR, and in situ diver investigation.

In the third sentence of the eighth paragraph under point 4 in the Taphonomic and depositional history subsection of the Results section, the correct sentence is: We interpret the available evidence to rule out the hypotheses arguing for wholesale secondary deposition given the state of current knowledge of coastal geomorphological and climatic processes, and rules in favour of the hypothesis that the underwater assemblage is largely in situ on a pre-inundation landscape and belongs to a much earlier time period and an entirely different pattern of landscape use than the archaeological site on the calcarenite terrace.

In the first sentence of the third paragraph of the Flying Foam Passage: A submerged freshwater spring subsection of the Results section, the correct sentence is: Just how much weight of interpretation can rest on a single artefact is, of course an issue, but the single artefact is the result of limited diving time and the difficulties of identifying artefacts against the background noise of other rocks and obscuring biogenic surface growth, and the details above confirm our interpretation that this is an in situ specimen.

## Updated Author Information

The ORCID iDs are missing for the third and seventh authors. Please see the authors’ respective ORCID iDs here:

Author Jo McDonald’s ORCID iD is 0000-0002-2701-7406 (https://orcid.org/0000-0002-2701-7406)

Author Patrick Morrison’s ORCID iD is 0000-0003-0920-3405 (https://orcid.org/0000-0003-0920-3405)

Additionally, the contributions for author Patrick Morrison are updated as follows: Data curation, Formal analysis, Methodology, Investigation, Software, Visualization, Writing–original draft, Writing–review & editing.

## Supporting information

S1 File DHSC Supplemental Data. (ZIP)
