# Supplementary figures and images for: Aboriginal artefacts on the continental shelf reveal ancient drowned cultural landscapes in northwest Australia
Source: PLoS One. 2020 Jul 1;15(7):e0233912. doi: 10.1371/journal.pone.0233912 (PMC7329065; doi:10.1371/journal.pone.0233912)

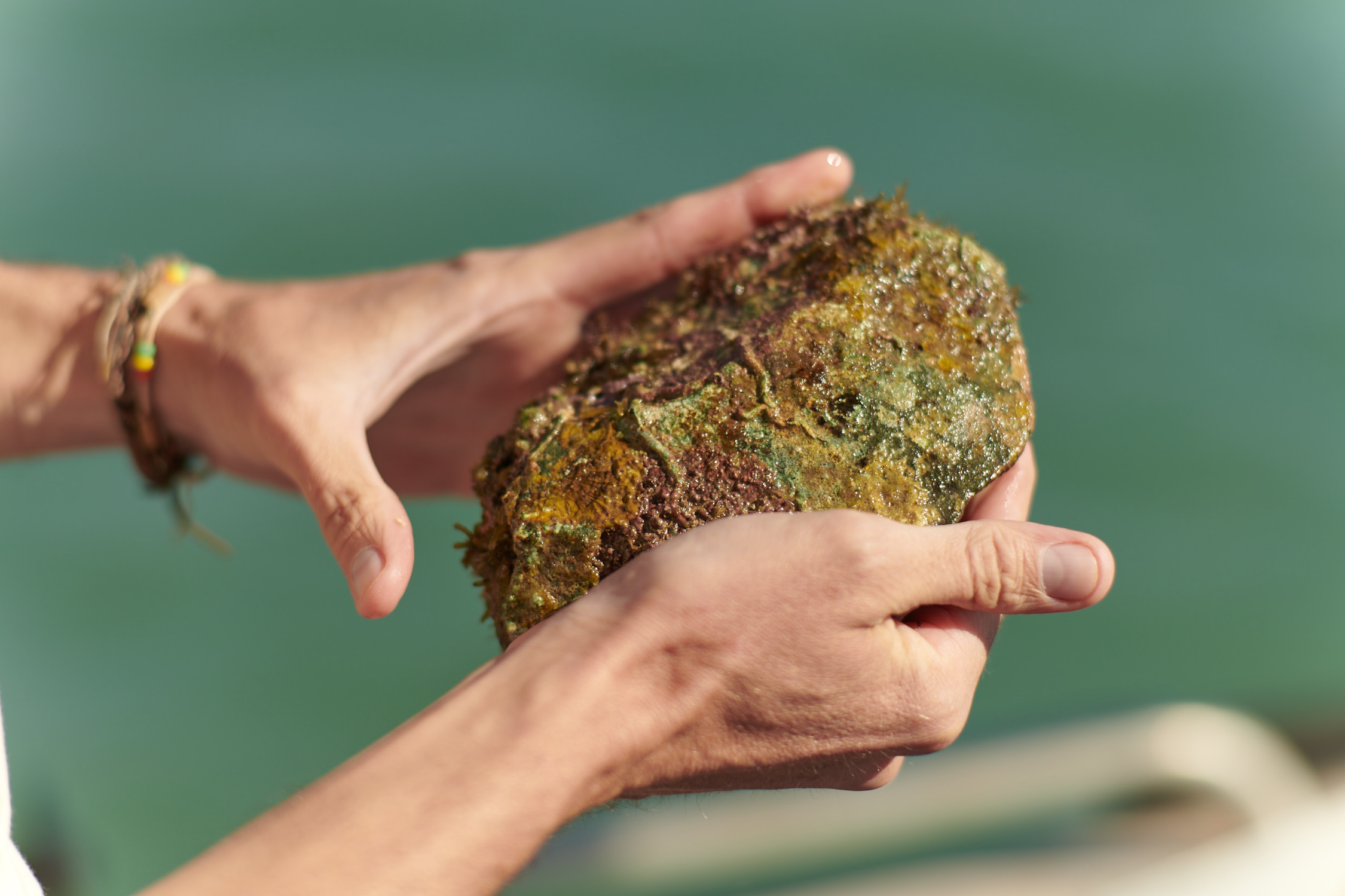

Supplement: S1 Fig — (TIF) [file pone.0233912.s001.tif]
