# Supplementary figures and images for: Correction: Aboriginal artefacts on the continental shelf reveal ancient drowned cultural landscapes in northwest Australia
Source: PLoS One. 2023 Jun 15;18(6):e0287490. doi: 10.1371/journal.pone.0287490 (PMC10270585; doi:10.1371/journal.pone.0287490)

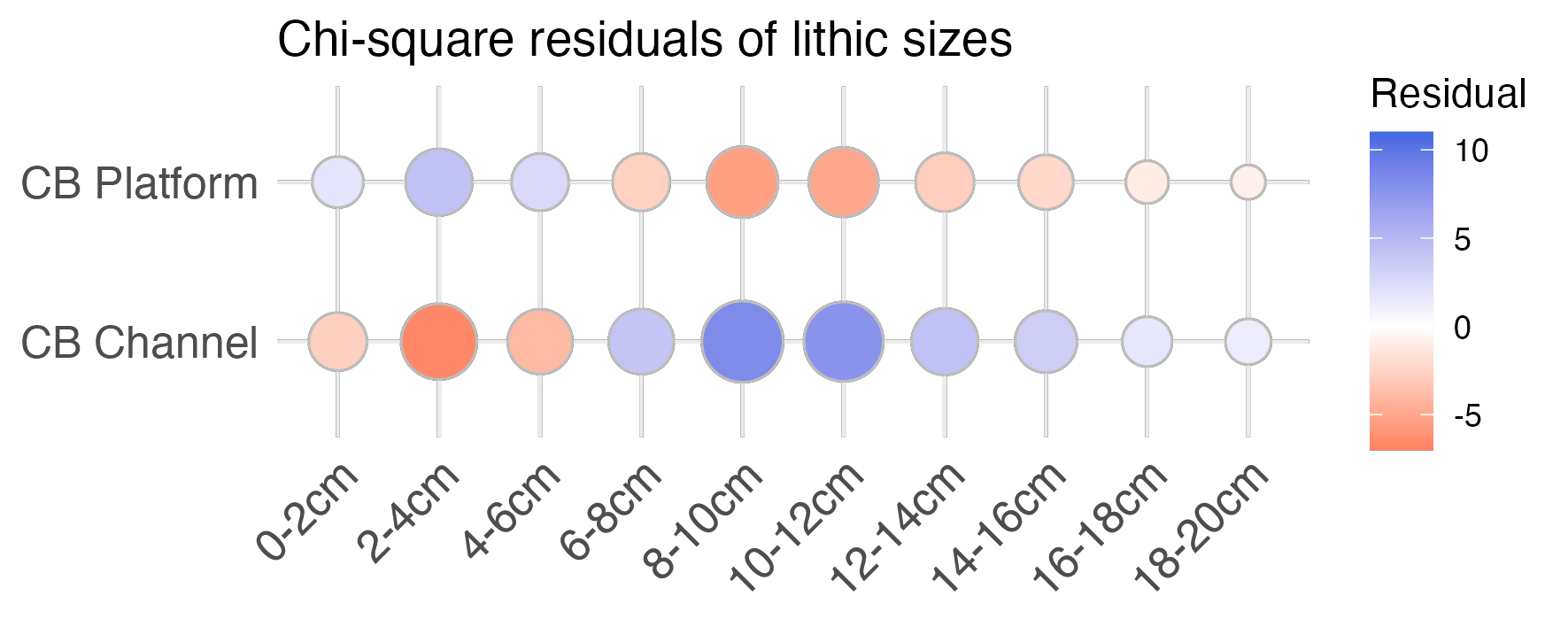

Supplement: S1 File — (ZIP) [file pone.0287490.s001.zip › S2_Fig.tiff]

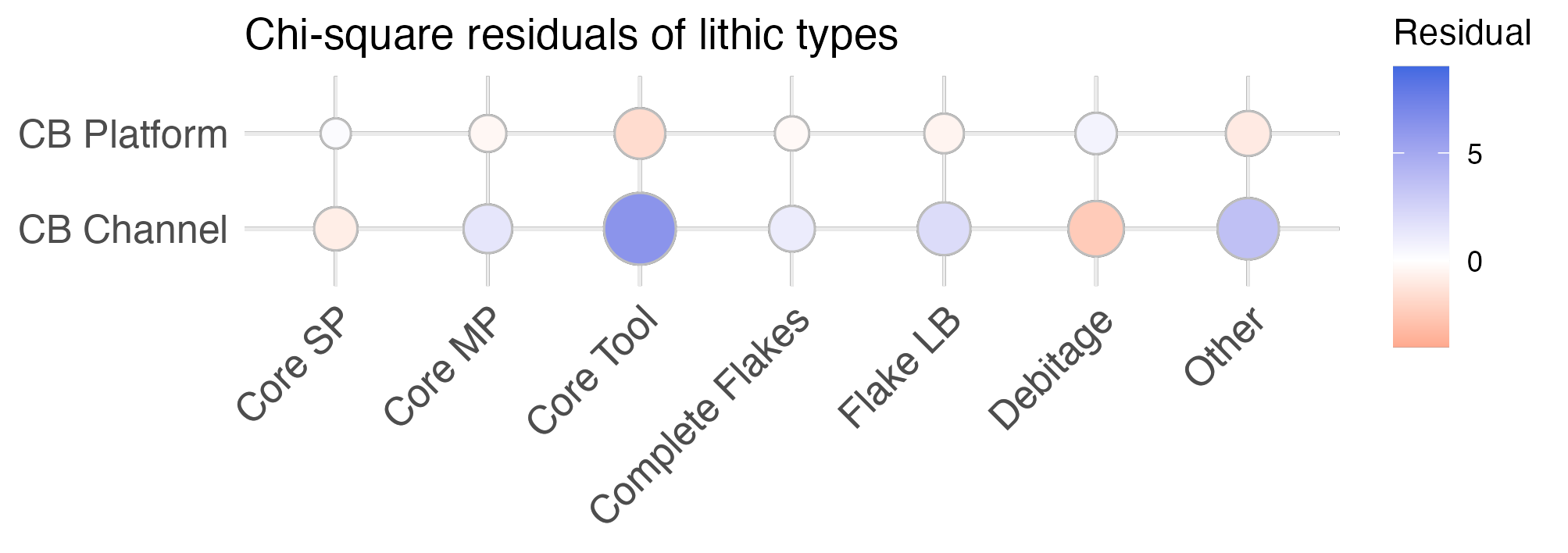

Supplement: S1 File — (ZIP) [file pone.0287490.s001.zip › S3_Fig.tiff]

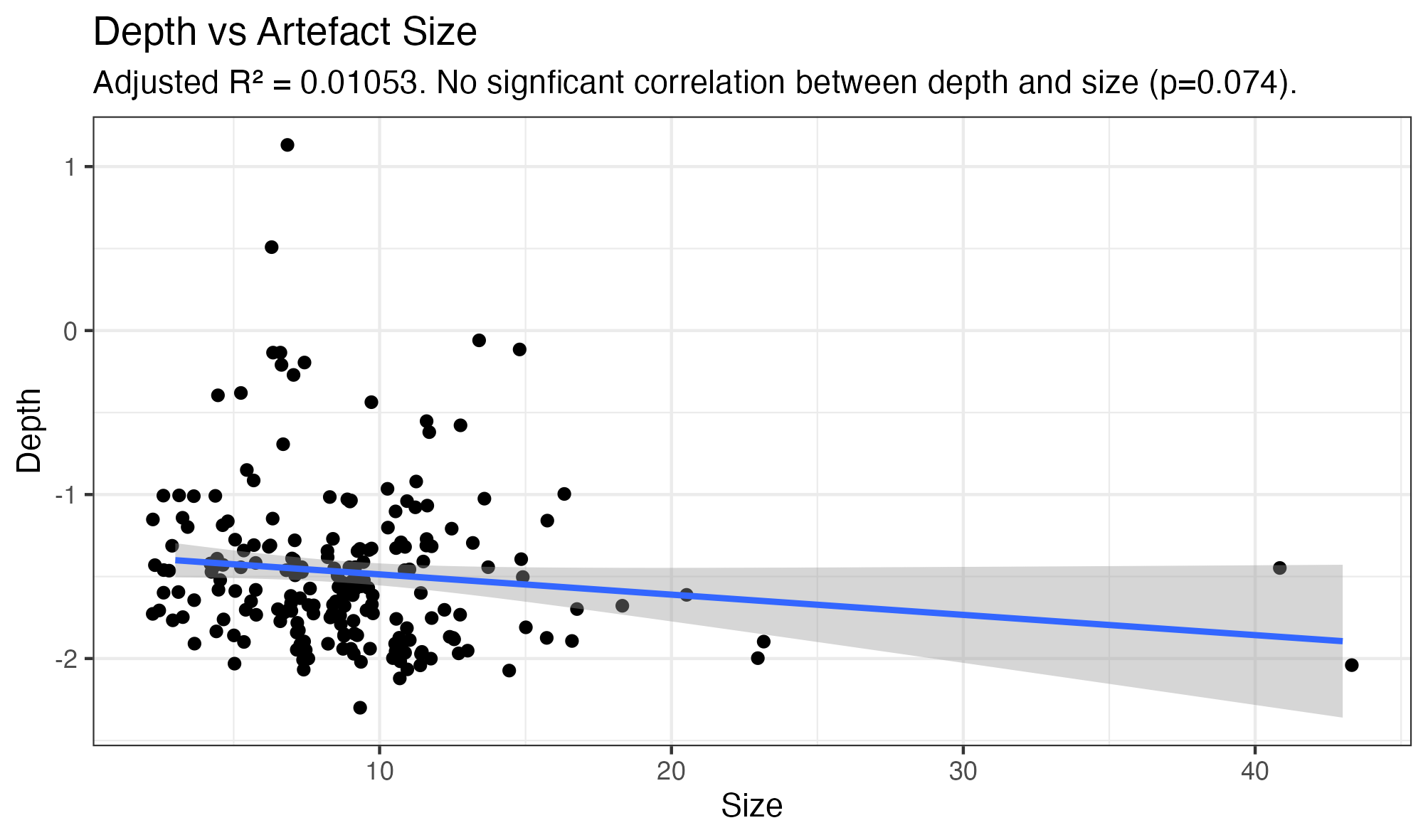

Supplement: S1 File — (ZIP) [file pone.0287490.s001.zip › S4_fig.tiff]

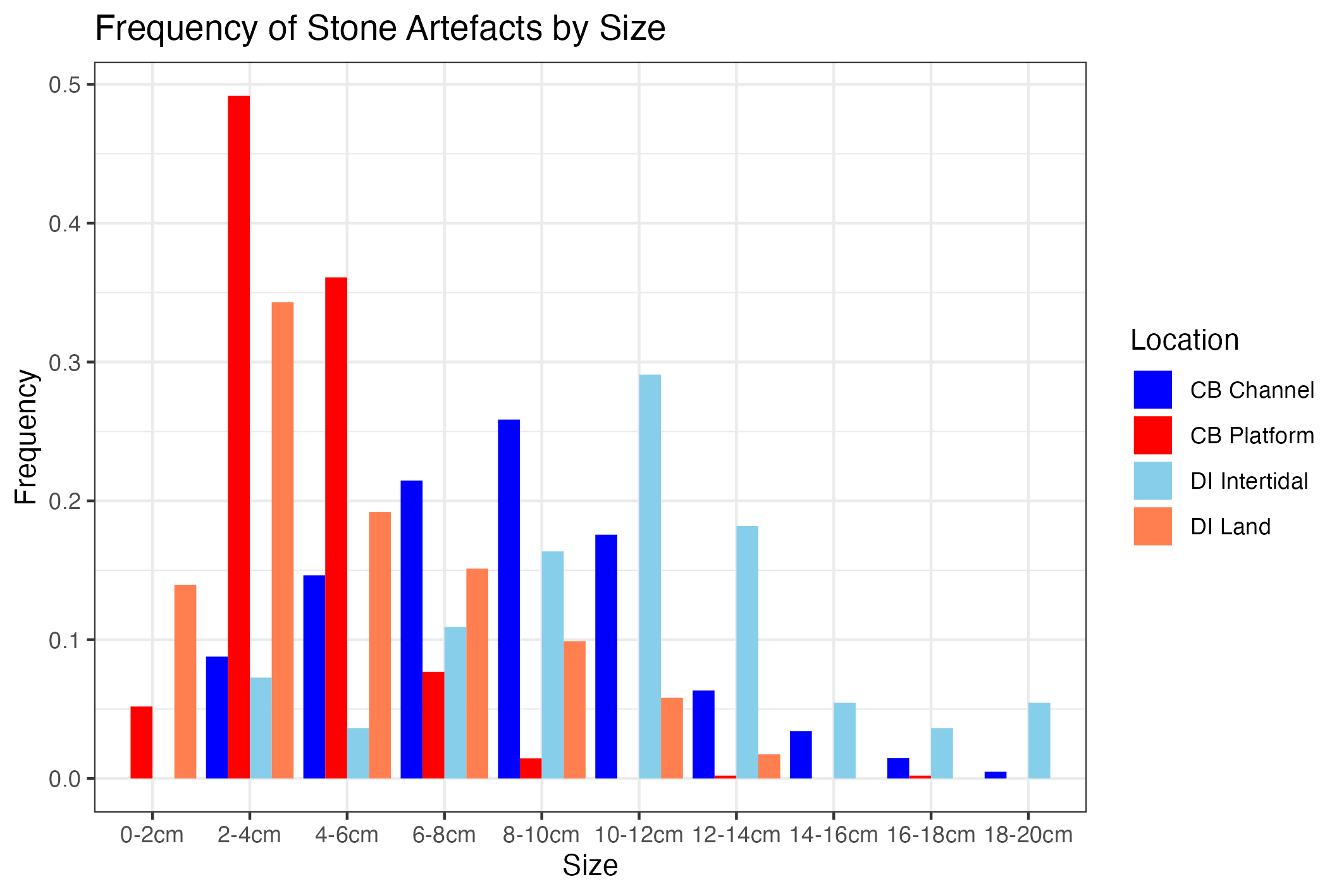

Supplement: S1 File — (ZIP) [file pone.0287490.s001.zip › S5_fig.tiff]

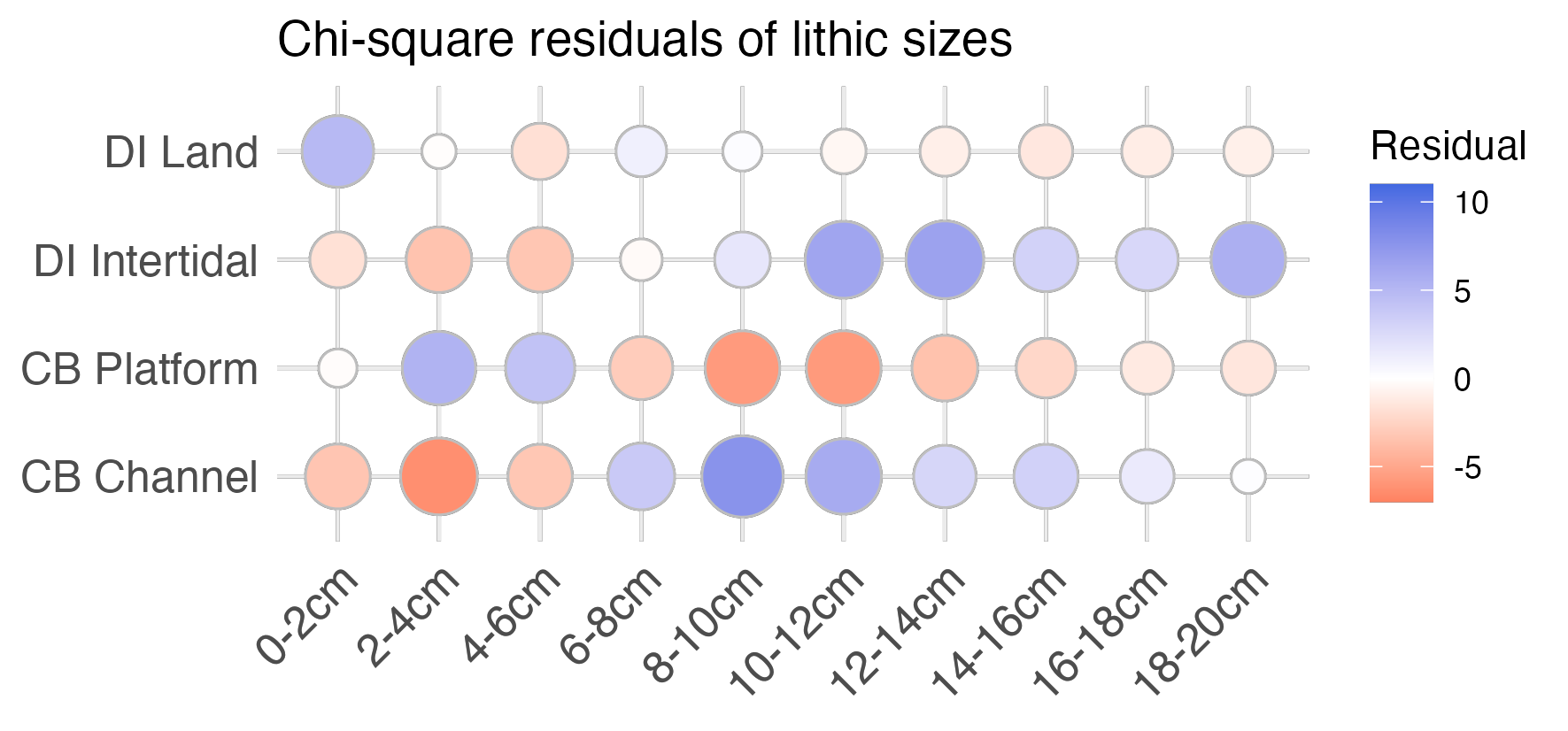

Supplement: S1 File — (ZIP) [file pone.0287490.s001.zip › S6_fig.tiff]
